# Taboo language across the globe: A multi-lab study

**DOI:** 10.3758/s13428-024-02376-6

**Published:** 2024-05-09

**Authors:** Simone Sulpizio, Fritz Günther, Linda Badan, Benjamin Basclain, Marc Brysbaert, Yuen Lai Chan, Laura Anna Ciaccio, Carolin Dudschig, Jon Andoni Duñabeitia, Fabio Fasoli, Ludovic Ferrand, Dušica Filipović Đurđević, Ernesto Guerra, Geoff Hollis, Remo Job, Khanitin Jornkokgoud, Hasibe Kahraman, Naledi Kgolo-Lotshwao, Sachiko Kinoshita, Julija Kos, Leslie Lee, Nala H. Lee, Ian Grant Mackenzie, Milica Manojlović, Christina Manouilidou, Mirko Martinic, Maria del Carmen Méndez, Ksenija Mišić, Natinee Na Chiangmai, Alexandre Nikolaev, Marina Oganyan, Patrice Rusconi, Giuseppe Samo, Chi-shing Tse, Chris Westbury, Peera Wongupparaj, Melvin J. Yap, Marco Marelli

**Affiliations:** 1 Department of Psychology, University of Milano-Bicocca, Piazza dell’Ateneo Nuovo 1, 20126 Milan, Italy; 2 Milan Center for Neuroscience (NeuroMI), University of Milano-Bicocca, Milan, Italy; 3 Department of Psychology, Humboldt-Universität zu Berlin, Unter den Linden 6, 10117 Berlin, Germany; 4 Department of Humanities, University of Trento, Trento, Italy; 5 School of Psychological Sciences, Macquarie University, Sydney, Australia; 6 Department of Experimental Psychology, Ghent University, Ghent, Belgium; 7 Department of Educational Psychology, The Chinese University of Hong Kong, Hong Kong, China; 8 Brain Language Laboratory, Department of Philosophy and Humanities, Freie Universität Berlin, Berlin, Germany; 9 Department of Psychology, University of Tübingen, Tübingen, Germany; 10 Centro de Investigación Nebrija en Cognición (CINC), Universidad Nebrija, Madrid, Spain; 11 School of Psychology, University of Surrey, Guildford, UK; 12 Centro de Investigação e Intervenção Social, Instituto Universitário de Lisboa, Lisbon, Portugal; 13 Laboratoire de Psychologie Sociale et Cognitive, CNRS, Université Clermont Auvergne, Clermont-Ferrand, France; 14 Department of Psychology, University of Belgrade, Belgrade, Serbia; 15 Center for Advanced Research in Education, Institute of Education, Universidad de Chile, Santiago, Chile; 16 Department of Computing Science, University of Alberta, Edmonton, Alberta Canada; 17 Department of Psychology and Cognitive Science, University of Trento, Trento, Italy; 18 Cognitive Science and Innovation Research Unit (CSIRU), College of Research Methodology and Cognitive Science, Burapha University, Chonburi, Thailand; 19 Faculty of Humanities, University of Botswana, Gaborone, Botswana; 20 Department of Comparative and General Linguistics, Faculty of Arts, University of Ljubljana, Ljubljana, Slovenia; 21 Department of English, Linguistics, & Theatre Studies, National University of Singapore, Singapore, Singapore; 22 Facultad de Filosofia y Letras I, Universidad de Alicante, Alicante, Spain; 23 School of Humanities, Foreign Languages and Translation Studies, University of Eastern Finland, Joensuu, Finland; 24 Department of Linguistics, University of Washington, Seattle, WA USA; 25 Department of Cognitive Sciences, Psychology, Education and Cultural Studies, University of Messina, Messina, Italy; 26 Department of Linguistics, Beijing Language and Culture University, Beijing, China; 27 Department of Psychology, University of Alberta, Edmonton, Canada; 28 Department of Psychology, Faculty of Humanities and Social Sciences, Burapha University, Chonburi, Thailand; 29 Department of Psychology, National University of Singapore, Singapore, Singapore

**Keywords:** Taboo words, Swearing, Semantics, Best–worst scaling, Emotion

## Abstract

The use of taboo words represents one of the most common and arguably universal linguistic behaviors, fulfilling a wide range of psychological and social functions. However, in the scientific literature, taboo language is poorly characterized, and how it is realized in different languages and populations remains largely unexplored. Here we provide a database of taboo words, collected from different linguistic communities (Study 1, *N* = 1046), along with their speaker-centered semantic characterization (Study 2, *N* = 455 for each of six rating dimensions), covering 13 languages and 17 countries from all five permanently inhabited continents. Our results show that, in all languages, taboo words are mainly characterized by extremely low valence and high arousal, and very low written frequency. However, a significant amount of cross-country variability in words’ tabooness and offensiveness proves the importance of community-specific sociocultural knowledge in the study of taboo language.

**Supplementary Information:**

The online version contains supplementary material available at 10.3758/s13428-024-02376-6.

Everyday communication is full of socially inappropriate words that are considered linguistic taboo. We are taught not to use them in conversation, even though we produce taboo words from the very moment we start speaking (Jay & Jay, 2013), and keep doing it throughout our lives. We also produce them while sleeping (Arnulf et al., 2017) or when acquired language disorders severely impair any other word production (Van Lancker & Cummings, 1999). As adults, 0.5% of the words we produce (i.e., ~80 words per day; Mehl et al., 2006) and 1% of the words we write on Twitter are taboo words (Wang et al., 2014). We use taboo words despite it being socially inappropriate, forbidden, and (in some countries) even legally punished. We do so because taboo language is an extremely powerful linguistic tool that fulfills an unparalleled wide range of psychological and social functions, as no other word category can do. Swearing allows us to induce emotional reactions (Sheidlower, 2009), insult others (Croom, 2011), increase the vividness of what is said (Azzaro, 2018), intensify emotional communication (Jay & Janschewitz, 2007), reinforce message effectiveness (Cavazza & Guidetti, 2014), increase the perceived credibility of the speaker (Rassin & Heijden, 2005), regulate emotions and reduce pain (Stephens & Umland, 2011), promote group bonding and reinforce group identity (Daly et al., 2004; Montagu, 2001), and elicit humor (Blake, 2018). Moreover, unlike all other words, taboo words (and in particular swear words) are used almost only with a connotative function (i.e., they do not refer to their literal meaning; Finkelstein, 2018; Jay & Janschewitz, 2008).

We do not all swear the same. Frequency of swearing is associated with personality traits (e.g., high scores of agreeableness and conscientiousness, as measured by the Big Five personality test, are associated with low frequency of swearing; Mehl et al., 2006), social factors (e.g., group identity; Daly et al., 2004), gender (men swear more frequently in public and use more offensive words than women; Jay, 2009), and idiosyncratic pragmatic factors, such as the conversational topic, the setting of the conversation (i.e., public/private, formal/informal), or the speaker–listener relationship (Jay & Janschewitz, 2008; Johnson & Lewis, 2010).

Studies investigating how taboo words are processed indicate peculiar properties of this category. Taboo words are remembered better than other words (MacKay et al., 2004), capture people’s attention (Carretié et al., 2008; MacKay et al., 2004), exert a detrimental effect on word recognition (e.g., Sulpizio et al., 2019) and speech production tasks (e.g., White et al., 2017), require a higher level of cognitive control (Dhooge & Hartsuiker, 2011; Scaltritti et al., 2021), increase the arousal level of the sympathetic nervous system (Harris et al., 2003; McGinnies, 1949), and persist in severe acquired language disorders hindering any other linguistic production (Van Lancker & Cummings, 1999).

Despite its wide use and relevance in fulfilling multiple social and psychological functions, we know very little about what taboo language is and what constitutes it across different populations, languages, and cultures. All our empirical knowledge on taboo language comes from a relatively small set of studies, almost entirely conducted in English and with limited cultural diversity. This poses two main theoretical problems. First, taboo language is highly conditioned by sociocultural factors, so what constitutes taboo can only be determined within a specific sociocultural environment. Hence, the currently available evidence offers an extremely restricted picture of the phenomenon. Second, because of this, even the composition, and thus the definition, of the taboo taxonomy is blurred. There is no agreement on the types and the number of categories characterizing taboo words (Jay, 2009; Stapleton, 2010). Finally, related to this last issue, it is still unclear what makes a word taboo. In terms of semantic properties, emotional aspects have been suggested to play a central role (Hansen et al., 2017; Jay & Jay, 2015). However, emotionality might not be enough to precisely characterize taboo words, which would otherwise be indistinguishable from other emotional words. Other properties that are typically considered are offensiveness (i.e., how a person perceives a word as inappropriate) and tabooness (i.e., how a person believes the society considers that word inappropriate; Jay, 1992). Nonetheless, while the use of the latter property makes the definition tautological, the former seems not to be a necessary property of swearing. For example, the English words *sex* or *vagina* are generally not offensive but are taboo in some social circumstances. In the data presented below, these are the words with the largest discrepancy between tabooness and offensiveness. The specific lexico-semantic characterization of taboo words is still to be determined, as it is still unknown whether and to what extent taboo words can be differentiated from non-taboo words on the basis of their lexical and semantic properties.

The present study aims at providing a first step towards filling these gaps, by collecting and characterizing taboo words in 17 different countries and 13 different languages (including some typically overlooked ones), covering all five permanently inhabited continents. In addition to offering a window into taboo language around the world, our study offers the unique chance to tease apart cross-linguistic from cross-cultural differences by analyzing the behavior of participants that speak country-based varieties of the same language (e.g., English in Canada and in Singapore). Importantly, taboo words in our study are defined in a strictly bottom-up manner based on speakers’ productions (Study 1). This allows us to establish what each community actually considers taboo without introducing any bias due to the researchers’ idiosyncrasies and normative definitions, and to identify commonalities and differences across languages and countries. In Study 2, we systematically collect intuitions about several semantic measures for each of the produced words to determine the combination of semantic features that best characterize the taboo dimension, and to evaluate their consistency across languages and cultures. Taken together, our results achieve two important goals: Theoretically, they contribute to a better general definition and understanding of taboo words and swearing across languages and cultures. Methodologically, they form a very rich database to study taboo language both per se and in relation to its several social and psychological functions.

## Study 1: Identifying taboo words

### Methods

#### Participants

We collected data in 18 labs from 17 countries (Australia, Belgium, Botswana, Canada, China [two labs, one in Beijing and one in Hong Kong], Chile, France, Germany, Finland, Italy, Serbia, Singapore, Slovenia, Spain, Thailand, United Kingdom, United States of America [US]), covering all five permanently inhabited continents and 13 different languages (Cantonese, Dutch, English, Finnish, French, German, Italian, Mandarin, Serbian, Setswana, Slovenian, Spanish, Thai), with some of these (i.e., English and Spanish) spoken in multiple countries. These languages are spoken as native language by more than 2 billion people in the world (i.e., ~25% of the global population, data from Wikipedia).

The total number of participants was 1046 (see Supplementary Table 1 for details), with each lab collecting data from at least 40 participants (40 to 167). Only native speakers of the language in question who lived in the country in question and who were not suffering from language-related and/or learning disabilities were included. Supplementary Table 1 reports participants’ details per lab as well as information concerning the ethics approvals obtained by each lab involved in the project.

#### Procedure

In each of the labs, a local coordinator managed all aspects of the study. The coordinator was a native speaker of the language in question living in the culture in which data collection occurred, or was flanked by another researcher who was a native speaker of the language in question and was living in the culture in which data collection occurred.

**Fig. 1:**
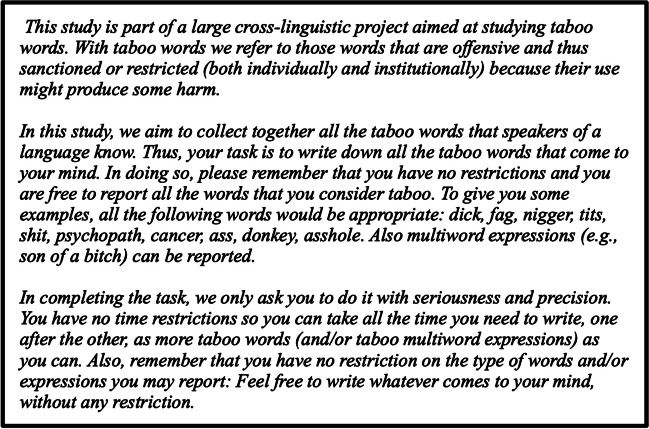
Instructions for Study 1

Participants were asked to freely write down all the taboo words they could think of. Both single-word and multi-word expressions were accepted, and examples were provided for both cases. There was neither time pressure nor any time restriction to complete the task. In the instructions, we specified that participants were free to write whatever came to their minds and encouraged them to avoid self-censorship. Instructions (in English) are reported in Fig. 1.

The instructions were provided to all the labs, which were asked to translate them in the local language and then back-translate them into English (translation and back-translation were not required for labs collecting data in English). Translation and back-translation were provided by different persons, and the back-translation was compared to the original version as a sanity check. Details about the data collection modality for each lab are reported in Supplementary Table 1.

For each sample, all participants’ productions were combined. In each lab, a researcher went through the list and (a) checked all the productions and corrected for possible minor errors (e.g., typos); (b) for non-English languages, provided an English translation of each word; and c) on the basis of their intuition as native speaker knowledgeable of the respective culture, classified each word (using a simplified taxonomy based on Jay, 2009) as belonging to one of the following categories for which definitions were provided to researchers: insult; slur; sexual; scatological referents and disgusting objects; profanities/blasphemies. When appropriate, researchers were invited to classify the same production in more than one category. When words could not be classified within the existing categories, researchers were allowed to create new categories. All annotated data are available at https://osf.io/ecr32.

Note that since there was no one speaker knowing *all* involved languages, we cannot guarantee that the very same classification and translation criteria were applied for all languages. Therefore, the information collected in (b) and (c) only provides a pointer to the word meaning, so that readers who do not speak the language have an opportunity to understand all the items in the dataset. However, we emphasize that this information should only be considered as a general reference and treated very carefully for any form of quantitative analysis.

#### Statistical considerations

In the linear mixed-effects model (LMM) analyses reported here, the 18 different samples served as our basic unit of observation; therefore, all LMMs reported here contain random intercepts for the samples in addition to the fixed effects specified in the individual analyses. We estimated the LMMs in R (R Core Team, 2022) using the packages *lme4* (Bates et al., 2015) and *lmerTest* (Kuznetsova et al., 2017).

### Results

**Fig. 2:**
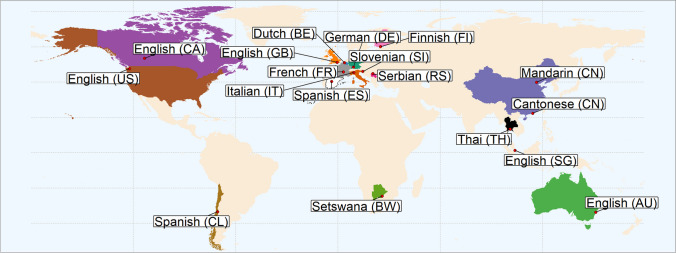
Map of the countries involved in Study 1 and Study 2: the 18 labs and the respective 17 countries and 13 languages in which data were collected

**Fig. 3:**
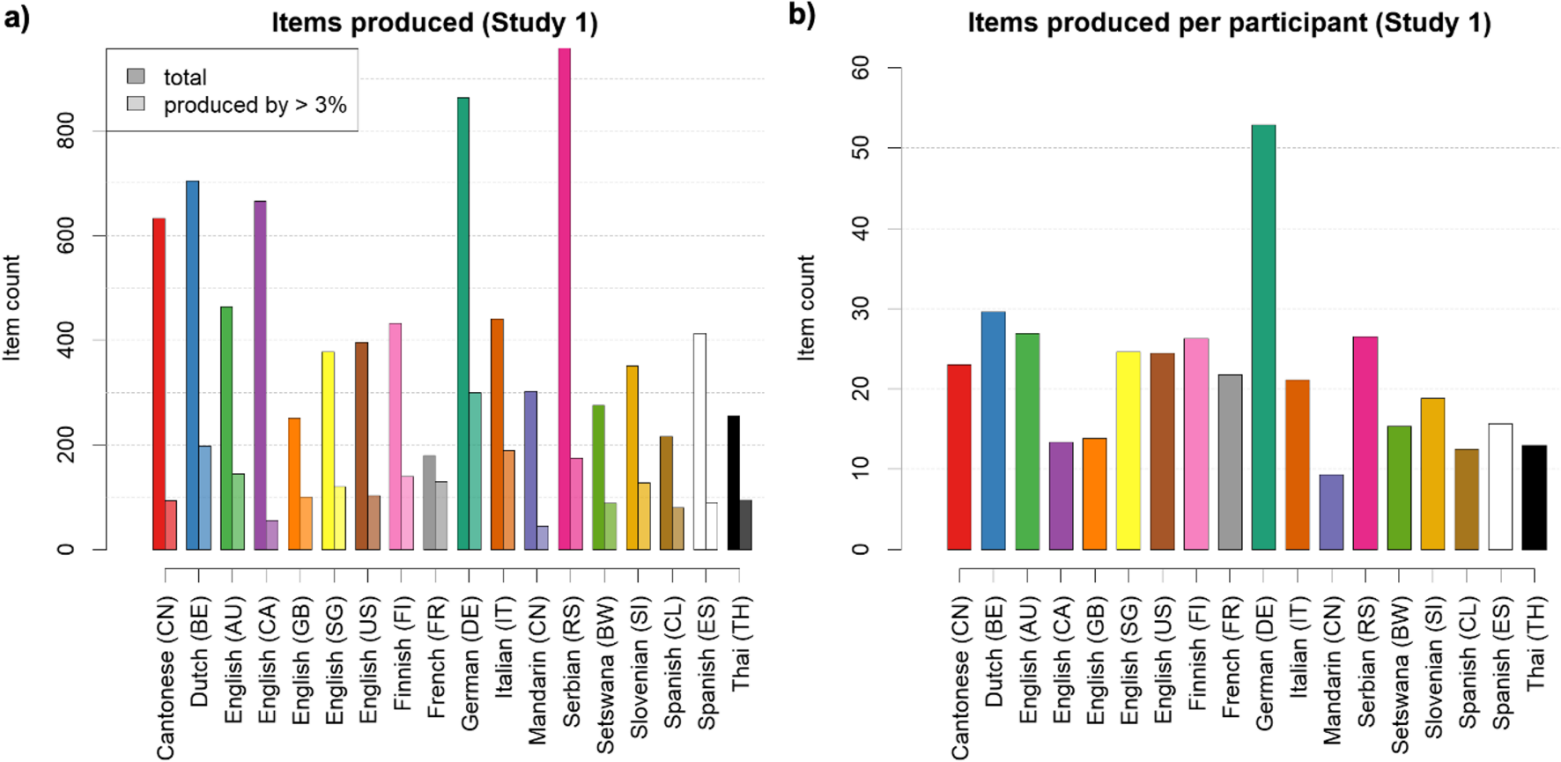
Number of items produced in Study 1 for each lab. **a** Total number of items (darker colors) and the subset of items produced by at least 3% of all participants (lighter colors). **b** Average number of items produced per participant

In Study 1, participants from 17 countries (see Fig. 2) were asked to freely generate any taboo words they could think of. The total number of words produced varies greatly between samples (see Fig. 3).

**Fig. 4:**
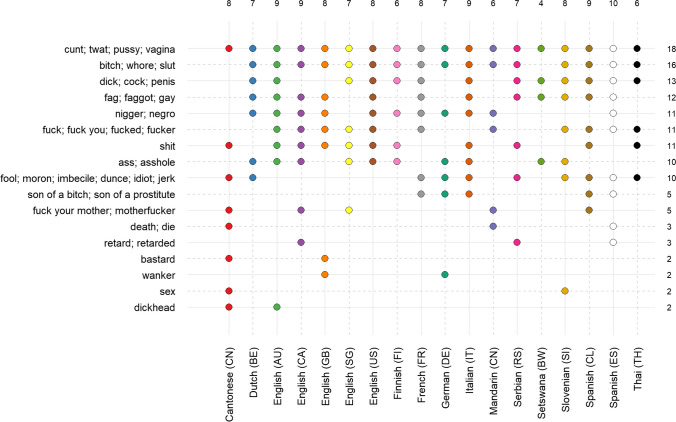
Samples in which a word is among the 10 most frequently produced ones. The figure shows the samples in which a word (or a semantically closely related word) is among the 10 most frequently produced words in Study 1, alongside the number of samples for which the word has been produced (right) and number of these items produced per sample (top). This figure only includes words appearing in the top 10 in at least two samples. Note that the taboo words reported in the figure refer to sets of meaning-related words (not exact translations) that were created on the basis of our intuition and should only be considered as qualitative

In a qualitative exploratory analysis, to assess the cross-language variability in our data, we manually inspected the 10 most frequently produced words in each sample and categorized them by means of their English translations (treating words with near-synonym translations as the same word). As can be seen in Fig. 4, there is a certain degree of consensus across samples: Some words are found among the most frequent words in many if not most languages. Variations of *cunt* (especially when also considering *mother’s cunt*) were seen in all samples, and those of *bitch* in almost all samples. Six additional items (*dick, faggot, nigger, fuck, shit,* and *ass*) were produced by about half of the samples. Also, with only a few exceptions, most samples produce around one third to a half of the 17 items (6–10 items), again suggesting some overlap in participants’ intuitions across languages and cultures. Note that almost *all* of these words are produced by some participants in every sample, but not frequently enough to appear among the ten most frequent words.

## Study 2: Evaluating taboo words

### Methods

#### Participants

Data were collected by the same labs for all the same languages and countries as in Study 1. The total number of participants was 455 for each of the six rating dimensions (see below), with the number of participants per lab depending on the length of their item list (see below). Only participants who self-declared to be in the 18–40 age range (the age range was fixed to this window as taboo language use may vary with age; Barbieri, 2008; Thelwall, 2008; note that this age range is the most typically used in the majority of psychological and cognitive science research), a native speaker of the language, and not suffering from language-related and/or learning disabilities were included. For each lab, approximately half of the participants self-identified as male, and the other half self-identified as female. Supplementary Table 2 reports all participants’ demographic information for each sample and rating dimension.

#### Materials

The taboo stimuli were based on the results of Study 1. We selected only those stimuli that were produced by at least 3% (rounded down) of participants (note that for samples up to 66 participants, this criterion is equivalent to only excluding *hapax legomena*, i.e., words that were produced by only one participant). Using a relative threshold allowed us both to account for between-samples variability in number of participants and to keep item sets manageable also for labs that had a large participant sample in Study 1.

To further keep the data collection manageable for all labs, the maximum number of taboo words to be included was set to 252. In cases where the procedure based on the 3% production criterion threshold produced less than 252 items, all taboo words above that threshold were included. On the other hand, in cases where the selection procedure based on the 3% production criterion threshold produced more than 252 words, the lab was required to apply a slightly more conservative threshold. For example, if following the 3% threshold rule and excluding words produced up to two times resulted in a list of 300 words, then the researchers were asked to exclude words produced up to three times. In cases where applying this slightly more conservative procedure then again produced a list shorter than 252 words, the researchers were required to fill the list up to 252 words by randomly sampling between the exceeding stimuli (i.e., from those that were produced between 3% and this more conservative threshold; see Supplementary Table 2 for the final number of selected taboo stimuli for each language; the stimuli for each language are available at https://osf.io/ecr32).

Taboo stimuli were presented along with filler non-taboo stimuli. The purpose of including filler words was three-fold: i) introducing variety in the item set to mitigate any list effect during the rating task (for example, avoid the lists to only include very negative words; this is especially relevant when using the best–worst paradigm to collect ratings, as described below); (ii) providing in the resource norms for non-taboo elements that can serve as matched control items for future studies; and (iii) allowing us to directly assess the external reliability of our ratings by comparing them to other existing word norms, which often do not include a large number of taboo words.

The total number of filler words was half of the number of taboo words eventually selected, rounded up. Filler words were selected to be representative examples of the language under investigation with respect to the two most important emotional dimensions, namely valence and arousal. Note that, as a representative sample, they are not matched to the taboo words, which we expect to score relatively higher on these dimensions—this expectation is confirmed in the analysis predicting the taboo word status of words (see the Results section of Study 2). Moreover, any a priori matching between taboo and filler words was impossible because of the lack of norms for taboo words in most of the languages under investigation.

To prepare the filler set, each coordinator was asked to rely on existing word norms. Where available, the adapted version of the Affective Norms for English Words (ANEW) database (Bradley & Lang, 1999) was used, and filler words were selected in order to have a distributional profile of valence and arousal (qualitatively) similar to that of the whole ANEW database. If the ANEW database was not available, the researchers could use any other database reporting information about valence and arousal and follow the same selection procedure described above. If no database with emotional dimensions was available for a given language, the English version of the ANEW database was used as inspiration, and then the selection was based on the intuition of a native-speaker researcher. In the case where the taboo stimuli included multi-word expressions, some filler multi-word expressions were also included (with the same proportion as the multi-word taboo expressions in the list). The list of filler stimuli for each language is available at https://osf.io/ecr32.

Instructions and debriefing information (in English, see Supplementary methods) were provided to all the labs, which were asked to translate them into the local language and then back-translate them into English (translation and back-translation were not required for labs collecting data in English). Translation and back-translation were provided by different persons, and the back-translation compared to the original version served as a sanity check.

#### Procedure

We collected ratings on six dimensions:

- Age of acquisition (when a word was learned; Brysbaert & Biemiller, 2017)
- Concreteness (to what extent a word referent can be perceived with the senses; Brysbaert et al., 2014)
- Valence (how pleasant a word referent is; Warriner et al., 2013)
- Arousal (the amount of excitement evoked by a word referent; Warriner et al., 2013)
- Offensiveness (how offensive a rater finds the word personally; Janschewitz, 2008)
- Tabooness (how taboo a word is, defined as to what extent it is *not* acceptable to use it in most social situations; Janschewitz, 2008)

Rating data were collected using the best–worst scaling technique (Hollis, 2018; Hollis & Westbury, 2018). In best–worst scaling studies, participants are presented with *N* items in each trial, and have to select the item which, in their opinion, scores the highest and the item which scores the lowest on a given dimension (for example, the least and most offensive item in the presented list). This produces information for 2(*N* − 1) pairwise comparisons per trial (the “best” item versus all other items, and the “worst” item versus all other items). Presenting each item in many different constellations of such sets of *N* items provides implicit rank information, making it possible to induce a rating scale from these best–worst judgments (Hollis & Westbury, 2018).

The optimal choice in terms of data quality and data-collection efficiency has been identified as *N* = 6 items per trial (Hollis, 2020). For this reason, the total number of items for each lab had to be divisible by 6. In case this criterion was not naturally met by the selection of taboo and filler stimuli (see Materials section above), a few additional fillers were added until the criterion was met.

We collected 30 observations for each individual item (that is, each item was presented in 30 different sets of six items; see Hollis, 2018, Experiment 4), which is the sufficient amount for near-asymptotic elimination of measurement errors (Hollis & Westbury, 2018). We used the software provided by Hollis (2018) to optimally arrange the item lists into sets of six, in a no-repetition setup that avoids presenting a combination of two words in more than one set, if possible (see Hollis, 2018, Experiment 4). Note that each participant was thus presented with a unique list of item sets as their trials.

Since the task could be tiring for participants, we decided that each single data collection session should not exceed 15 minutes (which corresponds to about 45 trials). This meant that in a lab with 252 taboo words and 126 fillers, at least 42 data collection sessions (and thus, the same number of participants) per semantic dimension were needed (252 data collection sessions in total). For smaller item sets, this number decreases in a linear manner.

In each session, the participant only responded to one of the semantic dimensions (e.g., only arousal or only offensiveness). Participants could not participate in more than one session for the same dimension, but they could take part in several subsequent sessions, one for each semantic dimension (e.g., a participant was prevented to evaluate “valence” more than once, but could evaluate both “valence,” “arousal,” and “tabooness” in different sessions if they desired). This allowed us to collect data also in labs with limited access to eligible and willing participants (which can result from organizational, financial, or even political constraints). Participants were informed beforehand that the study would contain words that many people consider offensive or inappropriate, that they would be directly confronted with very harsh language and offensive material, and that they could stop the experiment at any time if feeling uncomfortable.

All data were collected in a web-based experiment using *jsPsych* (version 6.3.0; de Leeuw, 2015). All labs sent all the experimental materials (including translations of instructions, scales, and debriefing) to one of the authors (FG) who implemented the experiment and sent back to the labs a ready-to-use link for data collection. This guaranteed a standardization of the experimental procedure, which was the same for all the labs. All data collections were hosted on servers of the University of Tübingen (FG’s affiliation at the time). Details on data collection modality are reported in Supplementary Table 2.

#### Constructing the rating scales

For each dimension in each sample, we estimated rating scores for all items from the explicit best–worst judgments using software provided by Hollis (2018). As a result, the items received a rating score between 0 (always selected as “worst”) and 1 (always selected as “best”). Note that this has important implications for the interpretation of these rating scores: Rating scores are inherently relative to the item set for which they were obtained. For example, if there is a clear least offensive item in a list, this item will always receive a low score, even if the list only consists of highly offensive items. This is the reason why we included fillers words that were selected to be overall representative of a language. Thus, the rating scores should be treated like values that are standardized *within each language *(without being directly comparable *between languages*). In addition, note that also the age of acquisition data is bound between 0 and 1, rather than directly indicating the age at which the word was learned (however, as shown later, correlations between these age of acquisition (AoA) scores and traditional AoA ratings are very high, so these scores can be transformed into an actual age via linear regression).

The rating scales were constructed from the judgments using the value learning algorithm (Hollis, 2018). For each dimension in each sample, we applied this scaling algorithm (a) on the entire dataset, (b) on the data collected from female and male participants separately, and (c) on two equally sized subsamples of the data, sampled by randomly assigning half of the participants to each subsample, in order to compute a split-half reliability for the rating scales (Günther et al., 2023).

#### Internal and external reliabilities

As a first step, reliabilities for each rating dimension were estimated by splitting the participant sample into two random halves, scoring these halves independently, and computing the correlation between these two sets of scores (see Günther et al., 2023). For the rating dimensions of AoA, valence, tabooness, and offensiveness, these reliabilities were high (*r* > .80) for most samples. For the rating dimensions of arousal and concreteness, these reliabilities tended to be overall lower; however, there was considerable variation between samples (see Supplementary analyses and Supplementary Fig. 1 for details). Note that this is however not unique to our study; in particular, arousal reliabilities tend to be lower in other large-scale rating studies using a general vocabulary (Warriner et al., 2013).

We further assessed these reliabilities quantitatively by means of the LMM reported in the “Gender differences” section of the Results for Study 2. As noted there, this model contained a fixed-effect interaction between type of correlation (by gender vs. random split-half) and dimension rated, as well as a random intercept and correlation type random slopes for the samples. The factors *AoA* for dimension rated and *split-half reliability* for correlation type served as reference level for the model; thus, all main effects are interpreted as differences to these reference levels. The absolute differences between rating dimensions are significant (*F*(5,187) = 42.68, *p* < .001), with considerably lower values on the dimensions “arousal” (*b* = −.13, *t* = −4.05, *p* < .001) and “concreteness” (*b* = −.21, *t* = −6.58, *p* < .001) as compared to the model’s intercept (i.e., the combination of the two reference conditions) of *b* = .84. There were no significant effects for the other rating dimensions (offensiveness, *b* = 0.03, *t* = 1.09, *p* = .279; tabooness, *b* = 0.04, *t* = 1.22, *p* = .225; valence, *b* = −0.04, *t* = −1.15, *p* = .250).

In addition to these internal reliabilities, we estimated the external reliability of our rating scores as the correlation with word norms collected in 35 other studies (see Supplementary analyses for more details, including a full list of these norms, their shared dimensions with our dataset, and the number of shared items). Note that for some languages generally or some semantic dimensions in some languages specifically, no external sources were available. In these cases, our data (notably including the data for the filler items) provide a first resource that can serve as a reference point for future studies.

We observed that external reliabilities were not significantly lower than internal split-half reliabilities. We thus have no evidence to indicate that the participants performing our rating tasks diverged substantially from the participant samples from other studies, despite the differences in item set structure and rating task (see Supplementary analyses for more information).

#### Word frequencies

For each item in Study 2, we considered two distinct measures of word frequency. On the one hand, we considered the production frequency in phase 1 as the percentage of participants producing a given word in the respective sample (to account for differences in phase 1 participant sample sizes across labs). On the other hand, we considered a written text frequency (the standard variant of word frequency measure, derived from written text corpora). In order to obtain largely comparable measures for written text frequencies, we based our estimates on the WaCKy web corpus family (Kilgarriff et al., 2010) or, where available, the structurally very similar but larger TenTen corpus family (Jakubíček et al., 2013) on SketchEngine (Kilgarriff et al., 2014). SketchEngine also provides the possibility to extract frequencies for multi-word expressions. Where applicable and available, we selected subcorpora for a specific language variant (namely for American English, Australian English, British English, Canadian English, European Spanish, and Chilean Spanish). To accommodate for differences in corpus size, all word frequencies were measured as frequency per million tokens. In line with previous research on frequency effects, all written corpus frequencies were log-Laplace-transformed via log(freq + 1) for all analyses reported (Brysbaert & Diependaele, 2013).

#### Statistical considerations

Again, as reported for Study 1, the LMMs (as well as generalized linear mixed-effects models, GLMMs) reported here contain random intercepts for the samples in addition to the fixed effects specified in the individual analyses, unless specified otherwise.

### Results

In Study 2, using a best–worst scale (Hollis, 2018; Hollis & Westbury, 2018), participants were asked to evaluate taboo words as well as representative filler items (see Methods for details) on six dimensions: age of acquisition (AoA), concreteness, valence, arousal, offensiveness, and tabooness. We started by qualitatively examining the most taboo and offensive words in each sample to look for possible cross-language and cross-country similarities. Then, by means of multiple quantitative analyses, we identified the semantic features that best characterize the taboo dimension and evaluated their consistency across languages and cultures.

For this quantitative analysis, it needs to be considered that although the reliabilities of our rating scales are generally high, some individual reliability scores are also low (see Supplementary Fig. 1). Even though this introduces noise in our variables, the analyses presented here mainly serve to describe and illustrate our dataset. Therefore, we included low-reliability rating scales in these analyses. For follow-up studies using our resource to investigate specific theoretical questions, we however strongly recommend researchers to select subsets of our dataset that meet their criteria and requirements, to be careful if using languages with low reliability and, in this case, consider the possibility to collect further data.

#### Qualitative examination

To understand what makes a word taboo or offensive, we started with a qualitative examination and manually inspected the semantic content of the 20 most taboo and 20 most offensive words in each sample. Keeping in mind that any qualitative summary will inevitably paint a somewhat oversimplified picture, some patterns do clearly emerge in the data. There are two classes of words that raters in most samples consider particularly taboo or offensive: sex-related words and slurs.

In virtually all samples, sex-related words take a central position, including those referring to specific sexual acts (*ass fuck*, *blowjob*) or genitalia (*cunt, dick*). Especially prominent are words that include forms of sexual violence or abuse (*rape, pedophile*) or words of all aforementioned categories that involve family members, including sexual behavior towards the addressee’s family members as in *“I fuck your mother,*” sexual behavior of the other towards their family members as in *motherfucker*, the sexual behavior of their family members as in “*son of a bitch,”* or references towards their family members’ genitalia as in *“Your mother’s smelly cunt.*” While present in the highest-rated words across all samples, sex-related words dominate the lists in the East Asian (China, Singapore, Thailand), Slavic (Slovenia, Serbia), and Spanish-speaking samples (Chile, Spain). A remarkable pattern within this class of words is a severe gender bias. The list of the most taboo/offensive words is almost exclusively populated by words referring to female genitalia (*cunt*) or female family members (*motherfucker, “I fuck your mother,” “son of a bitch”*). While the male counterparts to some of these words are also produced (*dick, cock, “I fuck your father,”* words for male sex workers), their offensiveness/tabooness ratings are not as high as for the female versions, and they consequently do not appear in the top-20 lists discussed here.

The second major class of highly offensive and/or taboo words are slurs that mostly refer to race (*nigger, wog*), gender (*bitch*, *whore*), and gender/sexual orientation (*fag, tranny*). Across many samples, racial slurs tend to address Black and Jewish people, as well as relevant ethnic groups in the respective countries (for example, slurs for Mexican people in the US, Pakistani people in Great Britain [GB], or Arab people in Belgium). Notably, slurs take a very prominent position in the Anglosphere (Australia, Canada, Singapore, GB, US) and Central European countries (Belgium, France, Germany, Italy, Slovenia).

A third class of frequent slurs refer to a violation of societal group expectations. Sexist slurs (*bitch, whore*) refer to women’s sexual looseness that goes against traditional gender roles, while homophobic (*faggot, dyke*) and transphobic (*tranny*) slurs refer to the violation of heteronormative and cisgender expectations related to sexual orientation and gender identity, respectively.

Beyond these major classes of taboo words, words referring to mental and/or physical disabilities (*retard*, *spastic*) are found in the most offensive/taboo words across most languages. Some other common types of words that appear across multiple different samples are death wishes, either directed at the addressees themselves (*“go die”*) or their family members (*“may your whole family die”* from Australia, Cantonese-speaking China, Belgium, and Spain), words referring to specific political ideologies (*Nazi* from Germany, France, Italy, and the US), or words suggesting low personal qualities of the addressee (*stupid, cheap type, ugly* from Cantonese-speaking China, Chile, Finland, Serbia, and Thailand). An interesting observation is that blasphemies (*fucking god, shit Christ*) only appear in the 20 most taboo and offensive words in Italy, while they are absent from all other lists inspected here.

#### Quantitative analyses

##### Gender differences

We first examined if there were structural differences when participant samples were split by gender (male vs. female) instead of randomly (which would point to systematic gender differences). To this end, we fitted a linear mixed-effects model (LMM) with a fixed-effect interaction between type of correlation (by gender vs. random split-half) and dimension rated, as well as a random intercept and by correlation-type random slopes for the samples. This is the same LMM as reported in the *Internal and external reliabilities* section in the Methods for Study 2. The levels *AoA* for dimension rated and *split-half reliability* for correlation type served as reference level for the model.

Across languages and rating dimensions, the correlations between male and female rating scores were comparable to the random split-half reliabilities. The fixed effect for correlation type was not significant (*b* = −0.13, *F*(1,187) = 2.97, *p* = .086), neither was its interaction with rating dimension (*F*(5,187) = 0.12, *p* = .988), indicating no structural gender differences beyond the expected random variation between any two groups of raters. Note, however, that the rating scores obtained with the best–worst scale are inherently relative to the item set for which they were obtained and thus do not allow making direct absolute group comparisons concerning the *mean values* of these ratings.

##### Relations between the semantic dimensions and frequencies

**Fig. 5:**
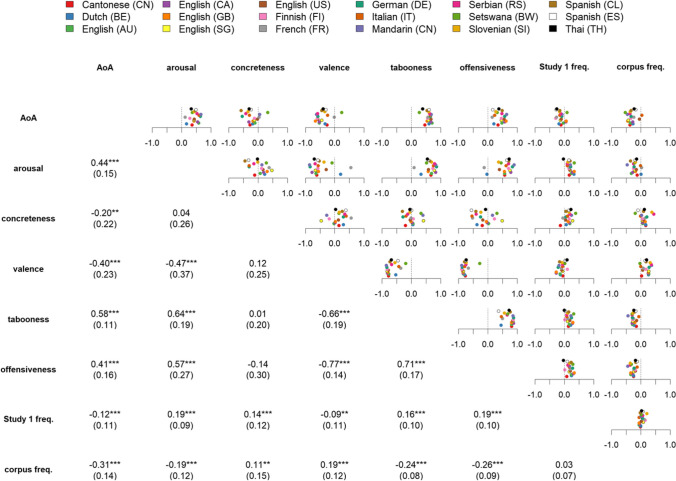
Correlations between rating dimensions and frequency measures. Pairwise Pearson correlations between all rating dimensions and the production frequency in Study 1 (*Study 1 freq.*) and the written corpus frequency (*corpus freq*.). The upper triangle shows correlation values for single samples (each sample represented by a colored circle); the lower triangle represents the means of these correlations with their standard deviation in parentheses. Mean correlations significantly different from zero (*p* < .001 in a *t*-test) are marked with ***

Figure 5 shows the pairwise Pearson correlations between semantic dimensions for the taboo words (i.e., without fillers) and (a) the production frequency from Study 1 and (b) the written corpus frequency. Some clear general trends can be observed across all labs (for example, a strong negative correlation between valence and offensiveness), but also a few cases with considerable variability (for example, when considering the correlation between concreteness and offensiveness).

First, it is worth noting that although there is some degree of association between offensiveness and tabooness, these variables are far from overlapping (mean *r* = .71). In addition, the two frequency variables show interesting patterns. First, the correlations between Study 1 production frequency and written corpus frequency are not significantly different from zero across all samples (mean *r* = .032, *t*(17) = 1.84, *p* = .083). While written word frequency is typically a good proxy for familiarity (Baayen et al., 2006; Balota & Chumbley, 1984), this relation clearly breaks down for taboo words. Taboo words produced more frequently in Study 1 are arguably those that speakers are more familiar with. However, these results indicate that a frequently produced taboo word in Study 1 is not frequently used in linguistic corpora. While one might suspect that participants in Study 1 refrained from producing very familiar taboo words because they are too offensive or taboo (i.e., self-censorship), this explanation appears unlikely given the consistently *positive* correlations between Study 1–production frequency and offensiveness and tabooness (see Fig. 5).

The relation between these variables and written frequency is the opposite. Tabooness and offensiveness show consistent negative correlations with written corpus frequency (see Fig. 5), indicating that “worse” words are less likely to appear in written language. This fits the very definition of tabooness. One is not supposed to produce taboo words in public language, and the written corpus frequencies were collected from publicly accessible sources. Thus, the dissociation between how written frequency and production frequency pattern with tabooness and offensiveness shows that speakers are aware of which words *should no*t be used in public language and tend to avoid using them, but *can easily* produce them when explicitly asked to. This is substantiated by data on word prevalence (i.e., the number of people knowing the word) showing that, among the 491 unique English taboo expressions of Study 1 present in Brysbaert et al.’s (2019) prevalence list, 86% are known by more than 90% of the speakers.

**Fig. 6:**
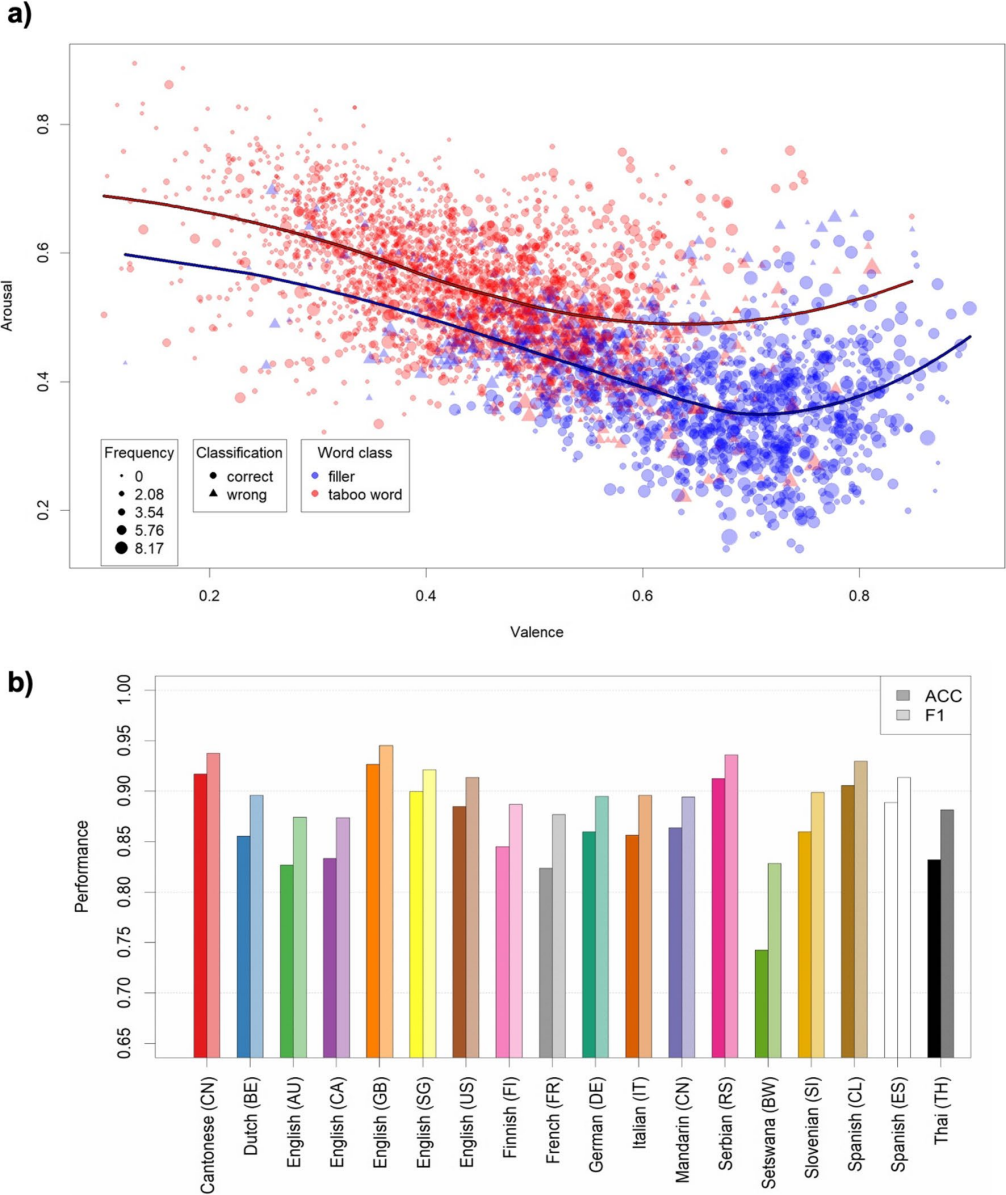
Illustrations of the categorical regression analyses predicting taboo word status from semantic variables and written corpus frequency. **a** Distribution of valence (*x*-axis), arousal (*y*-axis), and written corpus frequency (point size) for taboo words and fillers (color-coded) in the combined dataset of all samples, alongside their classification accuracy (point type). Regression lines predicting arousal from valence are fitted with local polynomial regression (loess) fitting. **b** Accuracy rates (darker colors) and F1 scores (lighter colors) for the LOOCV analysis, predicting taboo word status in the left-out sample with a GLMM trained on all other samples (including as predictors valence, arousal, concreteness, AoA ratings, and written corpus frequency)

In an additional analysis, we re-examined the prominent finding that the relation between the dimensions of valence and arousal is U-shaped rather than linear (Yik et al., 2023), to investigate whether this pattern holds for taboo words as well as non-taboo filler words across the wide variety of samples in our dataset. Although there is some heterogeneity across languages (see Supplementary analyses for details), overall, a U-shaped pattern indeed emerges for both classes of words across all samples (see Fig. 6).

##### What makes a word taboo/offensive?

Beyond exploring bivariate correlations, a main objective of our study was to test which lexical and semantic variables are systematically associated with tabooness and offensiveness. We investigated this question in three steps.

In the first step, we set up an LMM to predict the tabooness/offensiveness rating values of all words (taboo words and fillers) from the other rating dimensions (valence, arousal, concreteness, AoA) and written corpus frequency. Study 1 production frequency was not included as a predictor, because obtaining this variable requires instructing speakers to specifically produce taboo words, rendering it far less general than the other variables considered here. To further investigate dissociations between the tabooness and offensiveness dimensions, our LMM additionally included interaction terms between each of the described fixed effect predictors and a dummy variable coding for tabooness vs. offensiveness ratings (and a by-sample random slope for this dummy variable). The results of this analysis are displayed in Table 1.

**Table 1:** Results of the statistical models

		LMM predicting tabooness/offensiveness			GLMM predicting taboo word status		
Predictor type	Predictor	*b*	*t*	*p*	*b*	*z*	*p*
Intercept	Intercept	0.398	32.43	< .001	1.834	3.43	< .001
Main effects	Dummy	0.349	20.15	< .001			
	Valence	−0.515	−50.77	< .001	−9.586	−19.08	< .001
	Arousal	0.496	40.68	< .001	13.111	19.65	< .001
	Concreteness	0.068	6.58	< .001	−1.322	−2.77	< .001
	AoA	0.181	20.04	< .001	−2.364	−5.81	< .001
	Corpus freq.	−0.009	−10.53	< .001	−0.309	−7.90	< .001
Interactions	Dumm*y*: Valence	−0.209	−14.38	< .001			
	Dumm*y*: Arousal	−0.142	−8.26	< .001			
	Dummy: Concreteness	−0.139	−9.55	< .001			
	Dummy: AoA	−0.200	−15.65	< .001			
	Dumm*y*: Corpus freq.	−0.003	−2.39	.017			
*LMM predicting tabooness/offensiveness*: predictors of tabooness and offensiveness ratings across samples for the dataset of all words (words produced in Study 1 and fillers). “Dummy” is a dummy variable coding for tabooness ratings (coded as 0, the reference condition) or offensiveness ratings (coded as 1); therefore, the intercept and main effects except “dummy” describe tabooness ratings, while the “dummy” effect and all interactions describe how offensiveness ratings differ from tabooness ratings. *GLMM predicting taboo word status*: predictors of taboo word status across labs (1: taboo, 0: filler)							

As can be seen in Table 1, the main predictors for tabooness are valence (higher tabooness ratings for lower valence) and arousal (higher tabooness ratings for higher arousal). In addition, tabooness ratings are higher for words that are more concrete, are learned later (higher AoA), and appear less often in written language. Moreover, the influence of all these predictors is different for offensiveness ratings. For these, we found stronger negative effects of valence and written corpus frequency, as well as weaker positive effects of arousal, concreteness (to the point where the effect is negative; *b* = −0.07,* t* = −6.93 for the main effect in the same model with offensiveness as the reference condition for “dimension”), and AoA (to the point where it is slightly negative; *b* = −0.02, *t* = −2.08 for the main effect when offensiveness is the reference condition). Since the variables identified here could just be the ones telling apart taboo from non-taboo words, we repeated the analysis only on taboo words. The same results emerged (see Supplementary analyses).

In a second step, to specifically identify the factors discriminating taboo words from non-taboo (filler) words, we set up a generalized linear mixed-effects model (GLMM) as a categorical regression model to predict the taboo vs. non-taboo status, using the same fixed effects of the previous models (valence, arousal, concreteness, and AoA ratings, as well as written corpus frequency). All fixed effects significantly predicted taboo word status (see Table 1). A word is more likely to be a taboo word for higher values of arousal, but less likely for higher values of all other dimensions. Note, however, that in a bivariate comparison, AoA ratings tend to be higher for taboo words than for fillers (*M* = .541 vs. *M* = .414); therefore, the negative effect of AoA is likely the result of collinearity with other predictors. In terms of effect size, the *z* parameters in Table 1 show that low valence and high arousal are by far the most relevant predictors of taboo word status, followed by low written corpus frequency (see Fig. 6). The results of this analysis are thus very similar to the analysis predicting the tabooness of words.

The accuracy of this GLMM in predicting taboo word status is very high (*ACC* = .863, significantly higher than the no information rate (NIR) = .664, *p* < .001; *F1* = .900^38^).^1^ This is mainly because the very low valence and very high arousal of taboo words lie considerably outside the regular range of non-taboo (filler) words (see Supplementary analyses and Supplementary Fig. 2). Thus, by neglecting or even explicitly excluding taboo words, standard word norms systematically exclude the low end of the valence dimension and the high end of the arousal dimension.

To reduce overfitting and to investigate how well the effects of these variables predicting taboo status generalize across samples, we replicated this analysis while employing a leave-one-out cross-validation (LOOCV) procedure. We estimated the GLMM on all samples except one and used the parameters of the resulting model to classify taboo words in the left-out sample. The accuracy rates in the left-out samples ranged from .750 (Setswana as left-out, *F1* = .832) to .927 (English (GB) as left-out, *F1* = .945) (see Fig. 6) and were all significantly higher than the NIRs (all *p*s < .001; Kuhn, 2008). Thus, the results generalize very well across samples.

##### Differences between varieties of the same language

There are some instances where we collected data for the same languages from different samples around the world (five variants of English and two variants of Spanish). Our data can therefore provide us with an opportunity to disentangle linguistic and sociocultural factors.

**Fig. 7:**
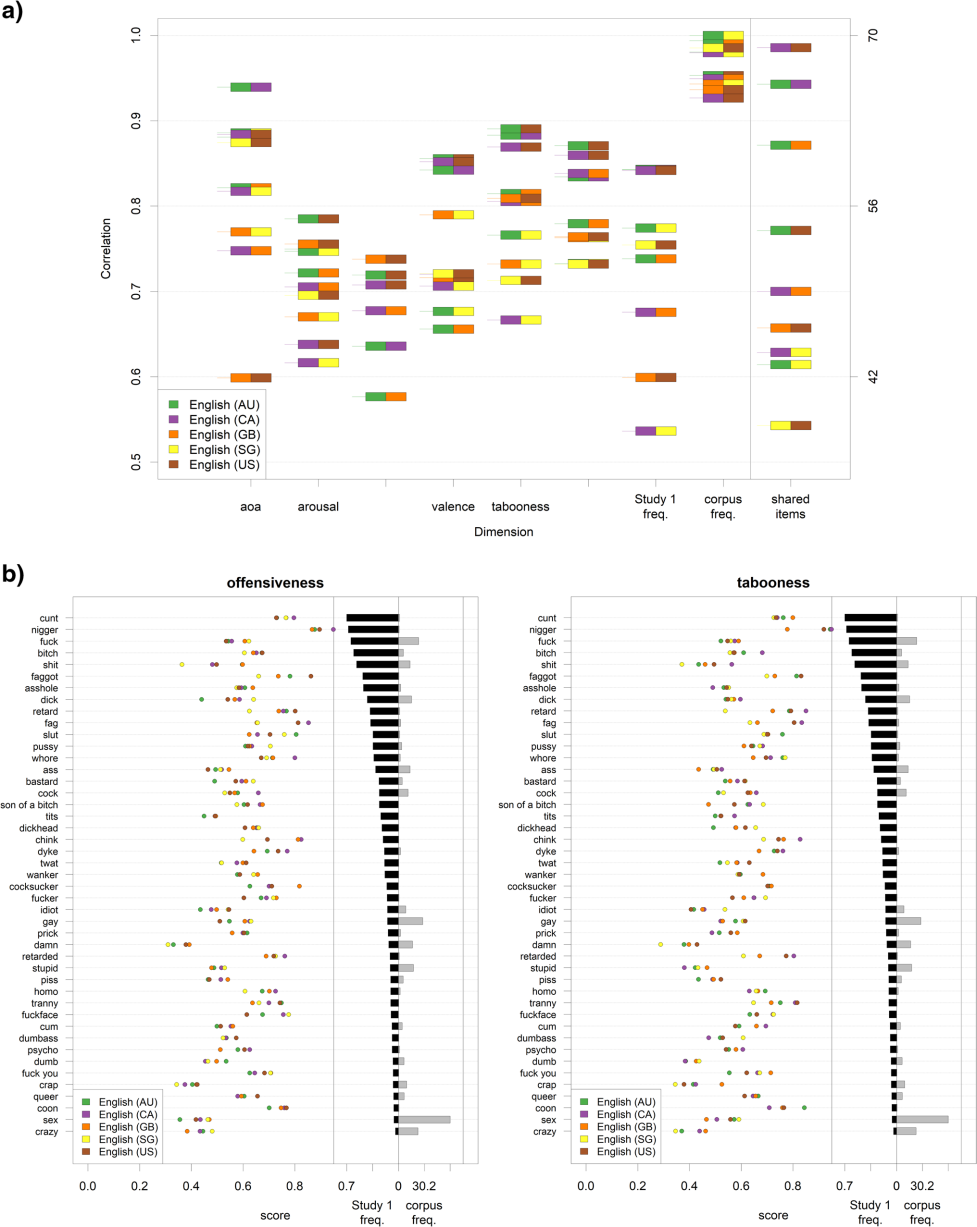
Differences and agreements between different varieties of English. **a** Left-hand side: correlations between the values on each rating dimension, Study 1 production frequency, and written frequency, for the different variants of the same language (English), computed on the shared items between these variants (AU: Australia; CA: Canada; GB: Great Britain; SG: Singapore; US: United States of America); the short horizontal lines indicate the correlation value between a pair of variants, the box next to the line indicates the pair of variants for which the correlation was computed. Right-hand side: the number of these shared items between pairs of variants. Note that the number of shared items is exactly the same for SG–US and SG–GB; thus, the latter is not visible in the plot. **b** Left-hand side of each plot: offensiveness (left plot)/tabooness (right plot) ratings by language variant for all the items appearing in at least four out of the five variants of English. Right-hand side of each plot: production frequency in Study 1 and written corpus frequency for these items (mean values)

**Fig. 8:**
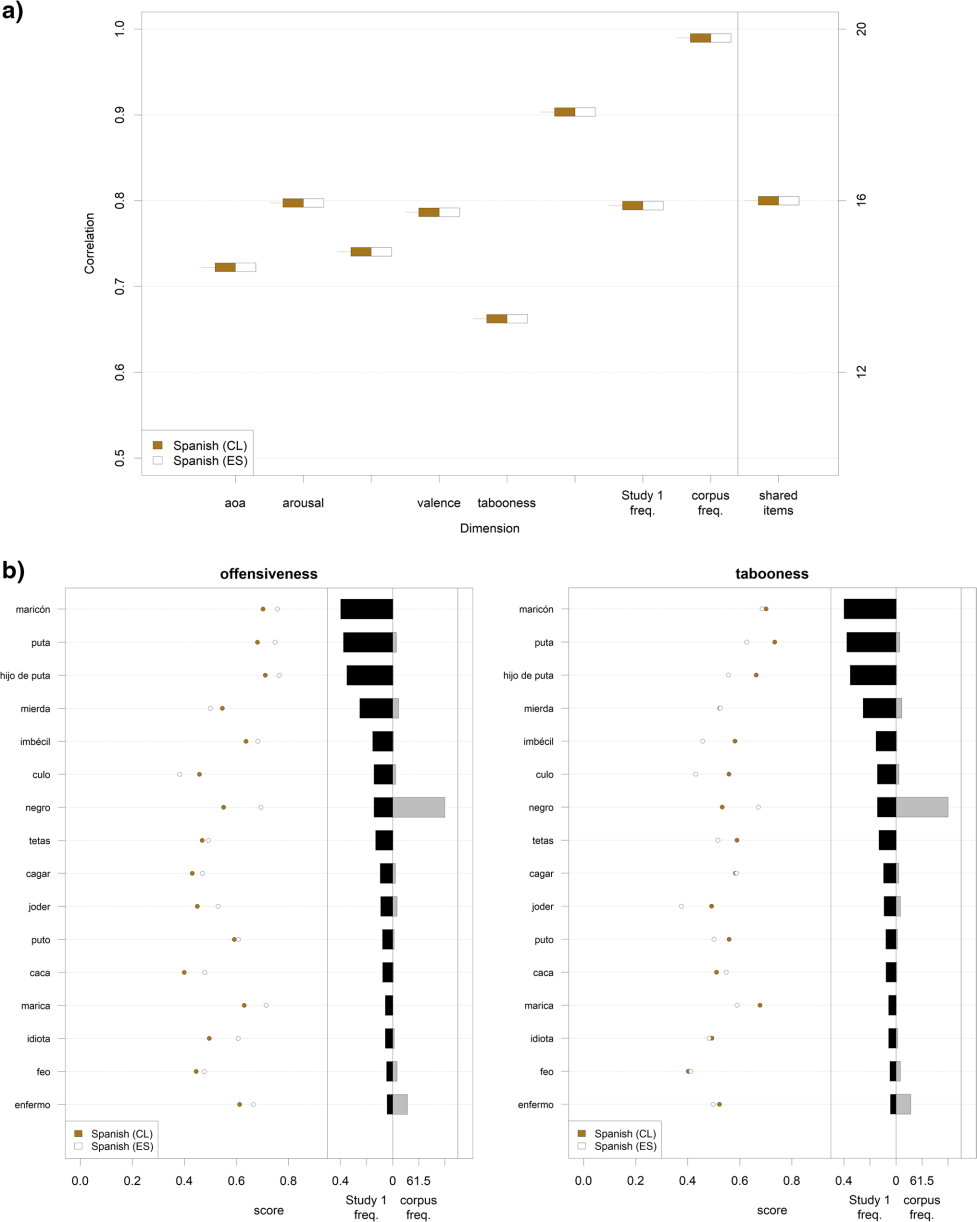
Differences and agreements between different groups of Spanish speakers. **a** Left-hand side: correlations between the values on each rating dimension, Study 1 production frequency, and written frequency for the two different variants of Spanish, computed on the shared items between these variants. Right-hand side: the number of these shared items. **b** Left-hand side of each plot: offensiveness (left plot)/tabooness (right plot) ratings by language variant for all items that appear in both variants of Spanish. Right-hand side of each plot: production frequency in Study 1 (black bars) and written corpus frequency (grey bars) for these items (mean values)

Figure 7 displays correlations between the different variants of English on the different rating dimensions, computed on their shared item sets (for the results on Spanish, see Fig. 8). For context, it should be kept in mind that upper limits to these correlations are posed by the reliabilities of these rating scores (see Supplementary analyses). Although the overall agreement is high, some variability emerges for all dimensions. Note, however, that these correlations are still relatively high in absolute terms (all > .70 for offensiveness, and > .65 for tabooness; all *p*s < .001).

A more fine-grained investigation allows us to explore which individual shared words are perceived similarly or differently across all five variants. Figures 7 and 8 exemplify how the content of the different types of taboo words is shaped by sociocultural sensitivity. Considering Fig. 7, for which data from more countries are available: Despite the first two words listed in the figure are those with the highest production frequency, some of them show a very high cross-country variability. For example, while *nigger* is perceived in all countries as extremely offensive in a similar way, it is considered less taboo in GB than in all other countries. On the contrary, *dick* is perceived as similarly taboo in all countries, but its offensiveness varies a lot. Finally, in some cases, there is an asymmetry between countries in the way a country ranks a word in terms of offensiveness and tabooness. This is the case, for example, for *retard*, which is more offensive but less taboo in the US than in Canada or Australia. Similar considerations also hold for Spanish (Fig. 8): *maricón* is similarly taboo in Spain and in Chile, but is considered more offensive in the former than in the latter; on the other hand, *puto* is similarly taboo in the two countries, but more offensive in Chile than in Spain.

### Discussion

The present study explored, for the first time, the taboo lexicon across several languages and countries, including some typically under-studied languages and populations. We provide a large resource to study taboo words. Moreover, our data offer a rich picture of what taboo language is and improve our knowledge about such a common linguistic behavior in at least three directions: (a) identifying what words are considered taboo and to what extent, (b) characterizing what makes a word taboo, and (c) dissociating the impact of culture to vis-à-vis language on the characterization of word tabooness and offensiveness (at least for English). In so doing, our results also contribute to better specify models of swearing (Senberg et al., 2021; Vingerhoets et al., 2013) by better characterizing contextual factors of swearing.

Differently from previous literature (Bertels et al., 2009; Janschewitz, 2008; Roest et al., 2018; Sulpizio et al., 2020), we have identified taboo words using a speaker-based (descriptive) rather than an expert-based (normative) approach. In this way, taboo words were defined on the basis of the intuition and the sensitivity of the linguistic community, in a way directly following from the definition of a taboo as a proscription of behavior for a specific community (Allan & Burridge, 2006; for a similar approach, see Jay, 1992). The results show that across samples, the category of taboo words ranges between ~50 and ~300 agreed-upon words, with most samples ranging between ~100 and ~200 words. Interestingly, amidst all the variation and differences among samples, there is also some considerable cross-language consistency on which words are seen as most taboo and offensive. Among the words considered the worst (and/or the most forbidden) by people all over the world, we found sex-related terms and slurs. Sex and sexuality have been considered taboo for a long time (Foucault, 1978) in many societies, arising from both social and self-proscription. Sexuality has to do not only with human body, but also with psychological, social, and moral dimensions (Weeks, 2011), which all may contribute to defining its tabooness. Slurs, on the other hand, explicitly target and degrade social groups (typically minorities). The fact that slurs were common among taboo words highlights the fact that such type of words are used to address individuals and groups “deviating” from traditional roles or norms. Importantly, slurs represent a sort of verbalized thought-crime (Nunberg, 2018) that can have consequences on interpersonal and intergroup relations (Fasoli et al., 2015, 2016). The use of slurs has thus relevant implications for psychological, social, ethical, and legislative dimensions (Council of Europe, 2021; Dzenis & Nobre Faria, 2020; Rosenblum et al., 2020).

Sex-related words and slurs show two interesting asymmetries. First, while the former tend to be more represented in the “worst words” in East Asian, Slavic, and Spanish-speaking countries, the latter tend to be more represented in Central European and English-speaking countries. Second, while a bias for sex words emerged such that they were mostly related to women words, slurs pointed to minorities and their “deviation” from social norms (i.e., women should not be sexually loose/promiscuous, people should be straight, people should be cisgender).

Of note, among the languages and countries we tested, although blasphemy has always been considered one of the main categories of taboo language (Jay, 1992), it is almost completely absent in our sample of “worst words,” and relatively infrequent in general. This is in line with diachronic linguistic analyses indicating that religion-related swear words are becoming more and more socially acceptable, especially in the English-speaking world (Mohr, 2013).

Within each language, taboo words were similarly ranked by males and females, suggesting that they perceive taboo words in a similar way. This is different from what was reported for emotional words (always using the best–worst scale; Mohammad, 2018). The absence of a gender difference, differently from language production, may be explained by the fact that everyone, regardless of their gender, has learned which words are taboo, as this is shared knowledge in a given society (Špago, 2020).

Our results allow us to characterize, from a psycholinguistic perspective, what makes a word taboo. Other than of course being characterized by high scores of tabooness and offensiveness (two dimensions that our data empirically show to be different constructs, and which we excluded from our analysis to predict taboo word status), taboo words are particularly related to valence and arousal—the more taboo and offensive words are also the more negative and highly arousing ones. In fact, the very low end of the valence distribution and the very high end of the arousal distribution are essentially only occupied by taboo words. Thus, by not considering taboo words in word norms or item lists, one will systematically ignore the far ends of the spectrum for these variables. At the same time, however, there is still considerable overlap between taboo and non-taboo words at the region of medium-to-high arousal and medium-to-low valence (cf. Fig. 6), demonstrating that the categorical distinction between taboo and non-taboo words cannot be based solely on these emotional dimensions. Such a distinction would require the assumption of a clear threshold, which is however absent in our data. Interestingly, tabooness and offensiveness are positively correlated with production frequency but negatively correlated with written frequency, the latter being the third most important predictor in the characterization of taboo words. This critical dissociation between the two frequency measures clearly shows that the most taboo words are those that everybody would consider as such and be familiar with, but that most speakers would avoid using in a regular public context. In this way, taboo words behave markedly in contrast to non-taboo words, for which written frequency is typically strongly associated with word familiarity (Baayen et al., 2006; Balota & Chumbley, 1984). This has important implications for corpus-based analyses and computational language models, which typically require a high number of observations to obtain meaningful observations and results. Taken together, valence, arousal, and written frequency are the dimensions mostly contributing to predicting tabooness and offensiveness (with smaller additional contributions of age of acquisition and concreteness), thus being largely sufficient to discriminate between a taboo and a non-taboo word. This emerges across all the samples we investigated (to the extent that taboo status in one sample can be predicted from a classifier trained on the other samples), suggesting that there is a common lexico-semantic characterization of taboo words across languages and cultures.

The availability of data from different countries speaking the same language allows us to dissociate the impact of culture to vis-à-vis language on the characterization of word tabooness and offensiveness. The presence of a significant amount of cross-country variability in these two dimensions (despite the fact that we are considering exactly the same words) is clear proof of cross-cultural variability and indicates that the study of taboo language cannot overlook sociocultural and pragmatics-specific knowledge to the community that is to be investigated. Interestingly, these data also offer a hint at the interaction between cross-country shared tendencies, detected at a superordinate lexico-semantic level, and sociocultural specificity, detected at a subordinate one. At the superordinate level, sex-related terms and slurs are considered taboo by all our samples. At the subordinate level, these two categories are filled according to sociocultural specific norms and sensitivities, showing a certain degree of variability in the category composition.

When used in actual communication, it is likely that all taboo words we report may be offensive, hurting, or violate social norms (after all, participants were explicitly instructed to produce exactly such words). Importantly, the general population might perceive these words as more taboo and offensive than reported here, as all our participants voluntarily produced or exposed themselves, and hence might not have been too bothered by taboo and offensive words to begin with. Therefore, our data cannot be used in any way to justify any violent and/or offensive behavior—low ratings on offensiveness or tabooness do *not* warrant or excuse the inappropriate use of these words and cannot serve as an argument that persons taking offense “are in the wrong” and that the words “aren’t so bad.” For the same reason, our results can be relevant for communication policies and strategies to monitor, detect, flag, and potentially prevent offensive linguistic behaviors in social network sites. We identified possible critical categories and words that require specific attention. Importantly, although some categories with cross-linguistic stability (as, e.g., slurs and sex-related terms) might be considered as a general guide for inspection and control, the critical contents should be specifically identified for each language with careful consideration of the words that do or do not harm that specific language community.

Before concluding, we believe is important to highlight some limitations of the present work. A first possible limitation refers to the way in which instructions of Study 1 were phrased, which might have biased participants in their productions. In particular, making explicit reference to offensiveness and giving some examples of taboo words might have either favored some categories (e.g., slurs and insults, which were represented among the examples given in the instructions) or penalized others (e.g., blasphemies, which were not present among the examples). Although we cannot exclude that instruction phrasing might have had an impact on which words were generated by participants, we believe this effect (if any) was limited. First, participants often generated words that were not mentioned in the instructions (e.g., blasphemies and political terms), suggesting that instructions did not substantially constrain participants in their generation; second, participants did not produce all taboo words provided in the instructions equally often (e.g., *donkey* was sporadically produced despite being one of the provided examples), suggesting that the words included in the instructions did not force participants’ productions. Future word-generation studies might try to test this issue by comparing the productions obtained in the present study with materials obtained through differently phrased instructions and/or offering different examples.

A second possible limitation of our study is that, although our data show a clear relation between tabooness and emotional dimensions, they are silent about causality. In other words, we do not know whether some words are taboo because they are highly negative and highly arousing, or the other way round. Although some data on this are available in the literature and seem to suggest that emotional connotation is a consequence of tabooness (i.e., taboo words are originally neutral and acquire emotional connotation because they are paired with punishment, e.g., Jay et al., 2006; Jay & Jay, 2013), the evidence is still scanty and mostly based on retrospective studies. Future research should tackle this issue by adopting longitudinal and/or experimental approaches so that a causal relation (if any) may be detected.

It is also worth noting that although this study offers an unprecedented set of data on taboo linguistic behaviors all around the world, it still neglects several languages typically unrepresented in the psychological literature. Although the issue might be hard to overcome because dealing with taboos is particularly problematic for some communities, we believe the present work represents an attempt to reduce the gap between the most researched languages and those that have been traditionally neglected, and may directly support future research on this topic by demonstrating its feasibility and relevance. Because of its rich psychological and sociocultural characterization, the use of taboo language has relevant implications for several fields such as psychology, linguistics, neuropsychology, and neuroscience, but also gender studies, sociology, and anthropology. Although more psycho-linguistically oriented, our results may be of relevance for all these disciplines and boost the study of taboo language, a possibly universal linguistic behavior.

### Supplementary Information

Below is the link to the electronic supplementary material.

Supplementary file1 (DOCX 441 KB)

## Data Availability

All materials, data, and scripts for the analyses are freely available at the link https://osf.io/ecr32/?view_only=60b964248cc64a8793a9013075132a1c.

## Footnotes

1 The F1 score is the harmonic mean of precision (the proportion of true positives in all positive classification results—how many identified items are relevant?) and recall (the proportion of true positives in all true cases—how many relevant items are identified?) for a binary classification.
