# Supplementary material for: Taboo language across the globe: A multi-lab study
Source: Behav Res Methods. 2024 May 9;56(4):3794–813. doi: 10.3758/s13428-024-02376-6 (PMC11133054; doi:10.3758/s13428-024-02376-6)
Supplement: Supplementary file 1 — Supplementary file1 (DOCX 441 KB) [file 13428_2024_2376_MOESM1_ESM.docx]

**Supplementary materials**

**Supplementary method**

***Study 1***

*Definition of the categories used to classify Study 1 productions*

*Insult*: an insolent/rude word/expression, that can offend and/or sound as an affront. Note that insults may refer to different types of (perceived or actual) deviations (physical, psychological, social; e.g., wimp, retarded). This category also includes some animal names that can be metaphorically used to refer to characterize people physical or psychological abilities (e.g., *donkey, monkey, pig*).

*Slur*: a pejorative term that targets people on the basis of their group (nationality, ethnicity, religion, gender, sexual orientation, etc; e.g., faggot, nigger).

*Sexual references*: any word/expression having as a referent a sex-related body part (e.g., cunt) or a sexual practice (e.g., *blow job*).

*Scatological referents and disgusting objects* (e.g., *crap, shit*).

*Profanities/blasphemies:* any irreverent word/expression toward/around God and/or sacred things (e.g., *goddamn*).

**Supplementary Table 1.** Summary of the Study 1 data collection – language, participants demographics, number of produced items, and ethical approval

| **Language** | **Recruitment, testing modality, reimbursement** | **N (male, female, other)** | **Age (mean, SD)** | **Items produced - unique types total (average per participant); produced by >3% of participants** | **Ethical approval** |
| --- | --- | --- | --- | --- | --- |
| Cantonese (CN) | University students, online, money | 41 (21, 20, 0) | 20.1 (1.2) | 632 (23.0); 93 | Survey and Behavioural Research Ethics, The Chinese University of Hong Kong; Reference number: EDU2020-098 |
| Spanish (CL) | Social media, online, none | 73 (5, 25, 0);  43 N.A. | 37.3 (5.9);  43 N.A. | 216 (12.5); 80 | Comité de Ética de la Investigación en Ciencias Sociales y Humanidades, Facultad de Filosofía y Humanidades, Universidad de Chile; Reference number 16/2020 |
| Dutch^[[1]](#footnote-1)^ (BE) | Prolific, online, money | 48 (-) | - | 704 (29.6); 198 | Ethical Committee Faculty of Psychology and Educational Sciences, University of Gent; Reference number 2020/115 |
| English (AU) | University students, online, course credit | 45 (14, 31, 0) | 22.2 (6.5) | 464 (26.9); 144 | Macquarie University Human Research Ethics Committee; Reference number #52020795419110 |
| English (CA) | Social media, online, none | 167 (69, 92, 6) | 39.5 (17.7) | 665 (13.3); 56 | University of Alberta REB; #Pro00115115 |
| English (GB) | University students, online, course credit | 59 (13, 46, 0) | 35.1 (12.3) | 251 (13.9); 100 | University of Surrey Ethics Committee; reference number FHMS 20-21 093 EGA |
| English (SG) | University students, online, money | 40 (20, 20, 0) | 25 (2.2) | 377 (24.6); 120 | Institutional Review Board, National University of Singapore; Reference Code: NUS-IRB-2021-2 |
| English (US) | Social media + Prolific, online, none or money | 71 (24, 42, 5) | 23.1 (5.5) | 396 (24.5); 103 | University of Washington Institutional Review Board; status Exempt, study id: STUDY00011426 |
| Finnish (FI) | University students, online, none | 49 (22, 27, 0) | 22.8 (1.7) | 432 (26.2); 139 | University of Helsinki Ethical Review Board in Humanities and Social and Behavioral Sciences; Statement 50/2021 |
| French (FR) | University students, pen and paper, course credit | 40 (13, 27, 0) | 22.6 (4.5) | 179 (21.7); 130 | Research Ethics Committee of Université Clermont Auvergne; Reference number IRB00011540-2020-51 |
| German (DE) | University students, online, money or course credit | 41 (21, 20, 0) | 22.02 (2.57) | 863 (52.9); 299 | Ethics committee for Psychological Research, University of Tübingen; Az Guenther_2020_0726_198 |
| Italian (IT) | University students, online, course credit | 62 (13, 48, 1) | 21.9 (1.1) | 441 (21.1); 189 | Committee for minimal risk Research Evaluation of the Department of Psychology, University of Trento; Protocol Number RM-2020-325 |
| Mandarin (CN) | University students, online, none | 44 (10, 33, 1) | 24.8 (3.4) | 301 (9.3); 44 | Ruled as “not required” |
| Serbian (RS) | Social media, online, personal contacts, none | 88 (19, 69, 0) | 25.8 (5.87) | 975 (26.5); 174 | Institutional Review Board of the Department of Psychology, University of Belgrade; Protocol #2020-43 |
| Setswana (BW) | University students + personal contacts, pen and paper, money | 45 (15, 30, 0) | 23.6 (6.2) | 275 (15.3); 88 | Office of Research and Development of the University of Botswana; reference UBR/RES/IRB/SOC/096 |
| Slovenian (SI) | University students + personal contacts, online, none | 43 (19, 24, 0) | 21.7 (2.5) | 352 (18.8); 127 | Ethical Committee Faculty of Arts, University of Ljubljana; Reference number: 209-2020 |
| Spanish (ES) | University students + personal contacts, online, none | 45 (17, 28, 0) | 29.6 (7.4) | 413 (15.6); 89 | Research Ethics Committee of Universidad Nebrija; Reference UNNE-2020-009 |
| Thai (TH) | University students, pen and paper, none | 45 (19, 24, 2) | 26.7 (6.8) | 254 (13.0); 94 | Burapha University Ethics committee (The SIDCER-FERCAP); Protocol number IRB2-016/2564 |

*Note: For each lab, the ethical approval encompassed both Study 1 and Study 2.*

***Study 2***

***Instruction for Study 2 data collection***

Thank you for participating in this study!

In the following study, you will be presented with sets of six words. There are no right or wrong answers, we are interested in your honest opinion. If you feel that several answers are possible, choose the one that best represents your opinion.

Note that some words can be offensive or insulting.

CONCRETENESS

Your task is to judge which of these words is the most concrete and which is the least concrete. A concrete word refers to something that exists in reality; you can have immediate experience of it through your senses (smelling, tasting, touching, hearing, seeing) and the actions you do. Therefore, “most concrete” refers to the word that you can experience the most using actions and senses, whereas “least concrete” refers to the word that you can experience the least using actions and senses.

AROUSAL

Your task is to judge which of these words is the most arousing and which is the least arousing. The most arousing word is the one evoking the strongest excitation or stimulation in you, no matter if good or bad. The least arousing is the word evoking the weakest excitation or stimulation in you, no matter if good or bad.

VALENCE

Your task is to judge which of these words is the most pleasant and the least pleasant. The most pleasant word is the one making you the happiest or more satisfied or more contented, whereas the least pleasant word is the one making you the most unhappy, unsatisfied, or sad.

AGE OF ACQUISITION

Your task is to judge which of these words you have learned or heard first in your life, and which you learned or heard at the latest point in your life.

TABOONESS

Your task is to judge which of these words is the most taboo and the least taboo one. Taboo means that the word use is not acceptable in most social situations. When completing the task, try to think about the use of the word in different contexts.

OFFENSIVENESS

Your task is to judge which of these words when used, is the most personally offensive and which is the least personally offensive.

*Debriefing of Study 2*

Thank you very much for your participation. The aim of this study is to assess words that are commonly considered as taboo words, namely socially inappropriate words, across different languages. This cross-linguistic project will examine taboo words used in more than 20 countries across the 5 continents. Despite their frequent use in everyday (oral and written) communication and their social relevance, taboo words have been almost neglected by empirical research on language. This project aims to start characterizing taboo words in many different languages. This will allow us to identify taboo words and empirically investigate how these taboo words are perceived and judged in multiple languages and cultures.

**Supplementary Table 2.** Summary of the Study 2 ratings – language, participants demographics, split-half reliability coefficients, and gender correlation

| **Language** | **Recruitment, testing modality, reimbursement** | **Number of items**  **(taboo, filler (superscript indicates the source for fillers))** | **Dimension** | **N (male, female, other)** | **Age (mean, SD, range)** | **Split-half reliability** | **Gender correlation** |
| --- | --- | --- | --- | --- | --- | --- | --- |
| Cantonese (CN) | University students, online, money | 144 (95, 49)  (Hutton & Bolton, 2005; Ng, 2006; 2010) | AoA | 16 (8, 8, 0) | 20.19 (1.83), 18 - 24 | 0.746 | 0.677 |
|  |  |  | arousal | 16 (8, 8, 0) | 20.31 (1.40), 18 - 23 | 0.857 | 0.744 |
|  |  |  | concreteness | 16 (8, 8, 0) | 20.31 (1.40), 18 - 22 | 0.736 | 0.676 |
|  |  |  | offensiveness | 16 (8, 8, 0) | 20.44 (1.82), 18 - 24 | 0.883 | 0.829 |
|  |  |  | tabooness | 16 (8, 8, 0) | 20.19 (1.28), 18 - 22 | 0.905 | 0.854 |
|  |  |  | valence | 16 (8, 8, 0) | 20.44 (1.82), 18 - 24 | 0.891 | 0.732 |
| Dutch (BE) | Prolific, online, money | 380 (249, 131)  (Moors et al., 2013) | AoA | 42 (21, 21, 0) | 25.43 (5.69), 18 - 40 | 0.886 | 0.883 |
|  |  |  | arousal | 42 (21, 21, 0) | 25.36 (5.93), 18 - 40 | 0.598 | 0.585 |
|  |  |  | concreteness | 42 (21, 21, 0) | 26.29 (5.87), 18 - 40 | 0.815 | 0.797 |
|  |  |  | offensiveness | 42 (21, 21, 0) | 26.14 (5.58), 19 - 39 | 0.857 | 0.859 |
|  |  |  | tabooness | 42 (21, 21, 0) | 25.52 (6.33), 18 - 40 | 0.889 | 0.885 |
|  |  |  | valence | 42 (21, 20, 1) | 25.67 (6.17), 18 - 40 | 0.884 | 0.877 |
| English (AU) | Prolific, online, money | 219 (149, 70) (Bradley & Lang, 1999) | AoA | 24 (13, 11, 0) | 21.54 (5.56), 18 - 36 | 0.847 | 0.873 |
|  |  |  | arousal | 25 (13, 12, 0) | 22.44 (5.73), 18 - 39 | 0.758 | 0.780 |
|  |  |  | concreteness | 25 (12, 13, 0) | 20.60 (4.11), 18 - 34 | 0.342 | 0.388 |
|  |  |  | offensiveness | 24 (11, 13, 0) | 19.54 (3.35), 18 - 30 | 0.877 | 0.847 |
|  |  |  | tabooness | 24 (11, 13, 0) | 19.67 (4.04), 18 - 38 | 0.904 | 0.905 |
|  |  |  | valence | 24 (12, 12, 0) | 21.17 (5.44), 18 - 38 | 0.861 | 0.853 |
| English (CA) | University students, in-lab, course credit | 378 (252, 126) (Hollis et al., 2017) | AoA | 42 (19, 20, 3) | 20.17 (3.62), 18 - 32 | 0.905 | 0.892 |
|  |  |  | arousal | 42 (16, 22, 4) | 20.45 (4.04), 18 - 37 | 0.862 | 0.819 |
|  |  |  | concreteness | 42 (22, 19, 1) | 19.55 (1.84), 18 - 25 | 0.640 | 0.608 |
|  |  |  | offensiveness | 42 (21, 21, 0) | 18.86 (3.07), 17 - 36 | 0.891 | 0.899 |
|  |  |  | tabooness | 41 (17, 22, 2) | 19.46 (2.46), 17 - 29 | 0.918 | 0.897 |
|  |  |  | valence | 42 (20, 20, 2) | 20.14 (2.65), 18 - 30 | 0.868 | 0.855 |
| English (GB) | Clickworker, online, money | 150 (100, 50) Bradley & Lang, 1999) | AoA | 18 (10, 8, 0) | 28.67 (6.97), 18 - 40 | 0.649 | 0.668 |
|  |  |  | arousal | 17 (8, 9, 0) | 31.71 (5.47), 20 - 38 | 0.371 | 0.305 |
|  |  |  | concreteness | 16 (7, 9, 0) | 33.44 (5.03), 25 - 40 | 0.548 | 0.412 |
|  |  |  | offensiveness | 17 (9, 8, 0) | 32.65 (5.65), 22 - 40 | 0.833 | 0.842 |
|  |  |  | tabooness | 16 (8, 8, 0) | 30.94 (6.27), 17 - 39 | 0.833 | 0.780 |
|  |  |  | valence | 18 (8, 10, 0) | 31.06 (5.77), 19 - 39 | 0.810 | 0.826 |
| English (SG) | University students, online, money | 179 (116, 63) (Warriner et al., 2017) | AoA | 20 (10, 10, 0) | 22.40 (1.93), 20 - 28 | 0.787 | 0.806 |
|  |  |  | arousal | 20 (10, 10, 0) | 22.30 (1.59), 19 - 26 | 0.786 | 0.771 |
|  |  |  | concreteness | 20 (10, 10, 0) | 21.95 (1.05), 20 - 24 | 0.441 | 0.507 |
|  |  |  | offensiveness | 20 (10, 10, 0) | 22.40 (1.90), 20 - 26 | 0.880 | 0.879 |
|  |  |  | tabooness | 20 (10, 10, 0) | 22.40 (1.67), 19 - 26 | 0.887 | 0.848 |
|  |  |  | valence | 20 (10, 10, 0) | 22.55 (2.21), 20 - 28 | 0.813 | 0.798 |
| English (US) | Prolific, online, money | 156 (102, 54) Bradley & Lang, 1999) | AoA | 18 (8, 10, 0) | 25.06 (6.76), 18 - 40 | 0.893 | 0.893 |
|  |  |  | arousal | 18 (7, 10, 1) | 27.39 (7.08), 18 - 40 | 0.760 | 0.788 |
|  |  |  | concreteness | 18 (8, 9, 1) | 26.33 (7.58), 18 - 40 | 0.577 | 0.621 |
|  |  |  | offensiveness | 18 (8, 9, 1) | 26.06 (7.67), 18 - 40 | 0.889 | 0.878 |
|  |  |  | tabooness | 18 (8, 9, 1) | 25.22 (6.42), 18 - 40 | 0.925 | 0.924 |
|  |  |  | valence | 18 (8, 9, 1) | 26.61 (6.43), 18 - 40 | 0.884 | 0.838 |
| Finnish (FI) | University students, online, none | 222 (148, 74) (Eilola & Havelka, 2010) | AoA | 13 (0, 0, 13) | 28.69 (7.08), 20 - 40 | 0.876 | 0.860 |
|  |  |  | arousal | 13 (0, 0, 13) | 27.92 (6.32), 20 - 38 | 0.717 | 0.636 |
|  |  |  | concreteness | 13 (1, 0, 12) | 26.62 (4.50), 23 - 38 | 0.791 | 0.775 |
|  |  |  | offensiveness | 13 (0, 0, 13) | 26.38 (7.03), 20 - 40 | 0.911 | 0.897 |
|  |  |  | tabooness | 13 (1, 0, 12) | 28.23 (6.27), 19 - 40 | 0.939 | 0.895 |
|  |  |  | valence | 14 (1, 0, 13) | 26.50 (6.45), 19 - 40 | 0.816 | 0.708 |
| French (FR) | University students, online, course credit | 204 (128, 76) (Monnier & Syssau, 2014) | AoA | 23 (12, 11, 0) | 19.48 (1.65), 18 - 23 | 0.909 | 0.883 |
|  |  |  | arousal | 23 (12, 11, 0) | 18.83 (1.27), 18 - 23 | 0.522 | 0.434 |
|  |  |  | concreteness | 23 (12, 11, 0) | 19.48 (1.97), 17 - 26 | 0.786 | 0.772 |
|  |  |  | offensiveness | 23 (11, 12, 0) | 19.52 (3.26), 17 - 30 | 0.913 | 0.900 |
|  |  |  | tabooness | 23 (11, 12, 0) | 21.96 (3.89), 18 - 30 | 0.913 | 0.854 |
|  |  |  | valence | 23 (11, 12, 0) | 22.48 (4.63), 18 - 33 | 0.868 | 0.815 |
| German (DE) | University students + Prolific, online, course credit or money | 378 (254, 124) (Kanske & Kotz, 2010) | AoA | 42 (20, 22, 0) | 24.31 (5.82), 18 - 37 | 0.885 | 0.865 |
|  |  |  | arousal | 42 (21, 21, 0) | 22.14 (3.49), 18 - 36 | 0.840 | 0.806 |
|  |  |  | concreteness | 42 (21, 21, 0) | 22.05 (3.60), 18 - 32 | 0.803 | 0.743 |
|  |  |  | offensiveness | 42 (23, 19, 0) | 23.31 (4.36), 18 - 36 | 0.909 | 0.895 |
|  |  |  | tabooness | 41 (20, 21, 0) | 23.34 (5.48), 18 - 40 | 0.905 | 0.915 |
|  |  |  | valence | 42 (22, 20, 0) | 22.95 (4.14), 18 - 40 | 0.898 | 0.882 |
| Italian (IT) | Prolific, online, money | 306 (212, 94) (Montefinese et al., 2014) | AoA | 32 (16, 16, 0) | 24.03 (4.12), 18 - 34 | 0.892 | 0.897 |
|  |  |  | arousal | 32 (16, 16, 0) | 25.56 (4.60), 19 - 38 | 0.852 | 0.834 |
|  |  |  | concreteness | 32 (16, 16, 0) | 24.62 (4.49), 18 - 37 | 0.808 | 0.812 |
|  |  |  | offensiveness | 32 (16, 16, 0) | 23.69 (4.72), 19 - 37 | 0.922 | 0.905 |
|  |  |  | tabooness | 32 (16, 16, 0) | 23.41 (3.28), 18 - 31 | 0.901 | 0.892 |
|  |  |  | valence | 32 (16, 16, 0) | 25.34 (4.63), 18 - 39 | 0.888 | 0.797 |
| Mandarin (CN) | University students, online, none | 66 (43, 23) (Lin & Yao, 2016) | AoA | 8 (4, 4, 0) | 29.38 (2.88), 25 - 33 | 0.772 | 0.806 |
|  |  |  | arousal | 8 (3, 5, 0) | 29.38 (2.72), 25 - 33 | 0.919 | 0.879 |
|  |  |  | concreteness | 8 (4, 4, 0) | 30.38 (3.78), 25 - 35 | 0.677 | 0.602 |
|  |  |  | offensiveness | 8 (3, 5, 0) | 29.50 (3.59), 25 - 34 | 0.897 | 0.894 |
|  |  |  | tabooness | 8 (3, 5, 0) | 30.00 (3.38), 25 - 35 | 0.886 | 0.847 |
|  |  |  | valence | 8 (4, 4, 0) | 31.12 (3.60), 25 - 35 | 0.838 | 0.897 |
| Serbian (RS) | University students + social media, online, none | 263 (174, 89) (Filipović Đurđević & Kostić, 2017; Popović Stijačić & Filipović Đurđević) | AoA | 30 (15, 15, 0) | 29.60 (7.35), 19 - 40 | 0.849 | 0.865 |
|  |  |  | arousal | 30 (16, 14, 0) | 28.50 (5.81), 18 - 40 | 0.853 | 0.841 |
|  |  |  | concreteness | 30 (15, 15, 0) | 28.87 (5.02), 20 - 40 | 0.760 | 0.695 |
|  |  |  | offensiveness | 30 (15, 15, 0) | 29.90 (6.47), 18 - 40 | 0.914 | 0.889 |
|  |  |  | tabooness | 30 (15, 15, 0) | 29.60 (6.75), 18 - 40 | 0.895 | 0.914 |
|  |  |  | valence | 30 (15, 15, 0) | 27.63 (6.28), 19 - 40 | 0.850 | 0.807 |
| Setswana (BW) | University students + personal contact, online + in-lab, money | 132 (87, 45) | AoA | 13 (6, 7, 0) | 21.23 (2.42), 19 - 28 | 0.753 | 0.625 |
|  |  | Fillers translated from Bradley and Lang (1999) | arousal | 15 (7, 8, 0) | 20.67 (1.45), 19 - 25 | 0.312 | 0.378 |
|  |  |  | concreteness | 15 (7, 8, 0) | 20.73 (1.62), 19 - 24 | 0.065 | -0.054 |
|  |  |  | offensiveness | 15 (7, 8, 0) | 22.93 (4.82), 19 - 37 | 0.671 | 0.658 |
|  |  |  | tabooness | 15 (7, 8, 0) | 21.67 (2.47), 19 - 28 | 0.664 | 0.588 |
|  |  |  | valence | 15 (8, 7, 0) | 22.33 (3.29), 19 - 30 | 0.014 | 0.190 |
| Slovenian (SI) | University students + personal contact + social media + Prolific, online, course credit or money | 378 (248, 130) | AoA | 42 (21, 21, 0) | 22.07 (2.99), 18 - 30 | 0.912 | 0.893 |
|  |  | Fillers translated from Bradley and Lang (1999) | arousal | 42 (20, 22, 0) | 22.52 (3.56), 18 - 30 | 0.696 | 0.716 |
|  |  |  | concreteness | 42 (20, 22, 0) | 22.07 (3.37), 19 - 30 | 0.676 | 0.522 |
|  |  |  | offensiveness | 42 (20, 21, 1) | 21.50 (4.63), 0 - 30 | 0.902 | 0.875 |
|  |  |  | tabooness | 42 (22, 20, 0) | 22.05 (3.28), 19 - 30 | 0.889 | 0.868 |
|  |  |  | valence | 42 (21, 21, 0) | 22.60 (3.58), 19 - 38 | 0.844 | 0.803 |
| Spanish (CL) | Social media, online, none | 180 (120, 60^15^) | AoA | 20 (12, 8, 0) | 23.35 (4.18), 18 - 32 | 0.867 | 0.851 |
|  |  |  | arousal | 20 (9, 11, 0) | 28.95 (6.13), 21 - 40 | 0.737 | 0.777 |
|  |  |  | concreteness | 20 (8, 12, 0) | 31.45 (6.23), 23 - 40 | 0.725 | 0.629 |
|  |  |  | offensiveness | 20 (9, 10, 1) | 27.50 (5.03), 20 - 39 | 0.864 | 0.849 |
|  |  |  | tabooness | 20 (10, 10, 0) | 30.40 (6.04), 21 - 40 | 0.886 | 0.883 |
|  |  |  | valence | 20 (11, 9, 0) | 30.45 (6.27), 21 - 39 | 0.837 | 0.820 |
| Spanish (ES) | University students + social media, online, none | 126 (82, 44) (Redondo et al., 2007) | AoA | 14 (7, 7, 0) | 22.86 (4.22), 19 - 32 | 0.909 | 0.915 |
|  |  |  | arousal | 14 (7, 7, 0) | 23.07 (4.10), 19 - 32 | 0.756 | 0.769 |
|  |  |  | concreteness | 14 (7, 7, 0) | 23.36 (4.29), 19 - 32 | 0.743 | 0.719 |
|  |  |  | offensiveness | 14 (7, 7, 0) | 24.29 (3.91), 19 - 32 | 0.890 | 0.874 |
|  |  |  | tabooness | 14 (7, 7, 0) | 22.79 (4.68), 19 - 32 | 0.849 | 0.883 |
|  |  |  | valence | 14 (7, 7, 0) | 23.57 (1.22), 22 - 26 | 0.830 | 0.784 |
| Thai (TH) | University students, online, none | 375 (254, 121) (Ngamprom et al., 2017) | AoA | 38 (16, 20, 2) | 24.39 (6.00), 18 - 40 | 0.818 | 0.771 |
|  |  |  | arousal | 37 (16, 19, 2) | 26.46 (6.73), 18 - 40 | 0.673 | 0.639 |
|  |  |  | concreteness | 36 (16, 17, 3) | 26.33 (6.20), 18 - 39 | 0.504 | 0.456 |
|  |  |  | offensiveness | 38 (16, 20, 2) | 26.63 (6.42), 18 - 39 | 0.866 | 0.855 |
|  |  |  | tabooness | 38 (19, 17, 2) | 26.16 (6.00), 17 - 39 | 0.855 | 0.874 |
|  |  |  | valence | 36 (17, 17, 2) | 25.47 (5.96), 16 - 39 | 0.809 | 0.773 |

**Supplementary analyses – Study 2**

***Calculating external reliabilities – correlations with other data sets***

To calculate external reliabilities, we collected all published word norms sharing the rating dimensions with our study. An overview of these word norms, alongside the number of shared items with our datasets and the shared rating dimensions, is provided in Supplementary Table 3. Note that we also considered some rating dimensions that only partially overlap with our dimensions, but share a very similar construct (*emotional charge* instead of *arousal* in Eilola and Havelka (2011); *imageability* instead of *concreteness* in the taboo word norms by Janschewitz (2008); *insult* instead of *offensiveness*, and *personal taboo* and *public taboo* instead of *tabooness* in the taboo norms by Roest and colleagues (2018)).

**Supplementary Table 3.** Overview of the word norms used in the current study for each sample to calculate the external reliability, together with the dimensions and the number of items shared with each resource used. Dashes (-) indicate cases where the word norms use slightly different terms than the ones established in our study, or additional specifications of these terms.

| **Language** | **Dataset** | **Shared dimensions** | **Shared items (taboo, filler)** |
| --- | --- | --- | --- |
| Cantonese (CN) | Cai et al. (2022) | AoA | 6 (6, 0) |
|  | Su et al. (2022) | AoA; concreteness | 11 (11, 0) |
|  | Xu and Li (2020) | concreteness | 9 (9, 0) |
|  | Xu et al. (2022) | arousal; valence | 10 (10, 0) |
|  | Yao et al. (2017) | arousal; concreteness; valence | 2 (2, 0) |
|  | Yee (2017) | arousal; concreteness; valence | 0 (0, 0) |
| Dutch (BE) | Brysbaert et al. (2014) | concreteness | 299 (173, 126) |
|  | Brysbaert et al. (2014) | AoA | 302 (176, 126) |
|  | Roest et al. (2018) | arousal; offensiveness - insulting; tabooness - general; tabooness - personal; valence | 89 (70, 19) |
|  | Moors et al. (2013) | AoA; arousal; valence | 192 (67, 125) |
| English (AU) | Bradley and Lang (1999) | arousal; valence | 94 (30, 64) |
|  | Brysbaert et al. (2014) | concreteness | 164 (97, 67) |
|  | Eilola and Havelka (2010) | arousal - emotional charge; concreteness; offensiveness; valence | 37 (26, 11) |
|  | Janschewitz (2008) | arousal; concreteness - imageability; offensiveness; tabooness; valence | 67 (48, 19) |
|  | Kuperman et al. (2012) | AoA | 162 (98, 64) |
|  | Warriner et al. (2013) | arousal; valence | 154 (89, 65) |
| English (CA) | Bradley and Lang (1999) | arousal; valence | 151 (28, 123) |
|  | Brysbaert et al.(2014) | concreteness | 265 (141, 124) |
|  | Eilola and Havelka (2010) | arousal - emotional charge; concreteness; offensiveness; valence | 40 (20, 20) |
|  | Janschewitz (2008) | arousal; concreteness - imageability; offensiveness; tabooness; valence | 95 (56, 39) |
|  | Kuperman et al. (2012) | AoA | 266 (143, 123) |
|  | Warriner et al.(2013) | arousal; valence | 247 (123, 124) |
| English (GB) | Bradley and Lang (1999) | arousal; valence | 59 (9, 50) |
|  | Brysbaert et al. (2014) | concreteness | 108 (58, 50) |
|  | Eilola and Havelka (2010) | arousal - emotional charge; concreteness; offensiveness; valence | 31 (23, 8) |
|  | Janschewitz (2008) | arousal; concreteness - imageability; offensiveness; tabooness; valence | 59 (37, 22) |
|  | Kuperman et al. (2012) | AoA | 109 (60, 49) |
|  | Warriner et al. (2013) | arousal; valence | 105 (55, 50) |
| English (SG) | Bradley and Lang (1999) | arousal; valence | 25 (13, 12) |
|  | Brysbaert et al. (2014) | concreteness | 84 (50, 34) |
|  | Eilola and Havelka (2010) | arousal - emotional charge; concreteness; offensiveness; valence | 19 (18, 1) |
|  | Janschewitz (2008) | arousal; concreteness - imageability; offensiveness; tabooness; valence | 37 (35, 2) |
|  | Kuperman et al. (2012) | AoA | 80 (49, 31) |
|  | Warriner et al.(2013) | arousal; valence | 88 (48, 40) |
| English (US) | Bradley and Lang (1999) | arousal; valence | 63 (11, 52) |
|  | Brysbaert et al. (2014) | concreteness | 115 (62, 53) |
|  | Eilola and Havelka (2010) | arousal - emotional charge; concreteness; offensiveness; valence | 31 (22, 9) |
|  | Janschewitz (2008) | arousal; concreteness - imageability; offensiveness; tabooness; valence | 58 (42, 16) |
|  | Kuperman et al. (2012) | AoA | 115 (65, 50) |
|  | Warriner et al. (2013) | arousal; valence | 114 (62, 52) |
| Finnish (FI) | Eilola and Havelka (2010) | arousal - emotional charge; concreteness; offensiveness; valence | 73 (14, 59) |
|  | Söderholm et al. (2013) | arousal; valence | 20 (0, 20) |
| French (FR) | Bonin et al. (2018) | arousal; concreteness; valence | 25 (7, 18) |
|  | Ferrand et al. (2008) | AoA | 21 (7, 14) |
|  | Monnier and Syssau (2014) | arousal; valence | 45 (1, 44) |
| German (DE) | Birchenough et al. (2017) | AoA | 84 (22, 62) |
|  | Kanske & Kotz (2010) | arousal; concreteness; valence | 159 (43, 116) |
|  | Schmidtke et al. (2014) | arousal; valence | 51 (19, 32) |
|  | Schröder et al. (2012) | AoA | 15 (10, 5) |
| Italian (IT) | Della Rosa et al. (2010) | AoA; concreteness | 17 (4, 13) |
|  | Montefinese et al. (2014) | arousal; concreteness; valence | 127 (35, 92) |
|  | Montefinese et al. (2019) | AoA | 131 (39, 92) |
| Mandarin (CN) | Cai et al. (2022) | AoA | 12 (8, 4) |
|  | Su et al. (2022) | AoA; concreteness | 9 (7, 2) |
|  | Xu and Li (2020) | concreteness | 11 (4, 7) |
|  | Xu et al. (2022) | arousal; valence | 13 (5, 8) |
|  | Yao et al. (2017) | arousal; concreteness; valence | 5 (3, 2) |
| Spanish (CL) | Alonso et al. (2015) | AoA | 52 (19, 33) |
|  | Guasch et al. (2016) | arousal; concreteness; valence | 12 (4, 8) |
|  | Hinojosa et al. (2016) | arousal; concreteness; valence | 1 (1, 0) |
|  | Redondo et al. (2007) | arousal; valence | 40 (7, 33) |
|  | Stadthagen-Gonzalez et al. (2017) | arousal; valence | 53 (20, 33) |
| Spanish (ES) | Alonso et al. (2015) | AoA | 66 (35, 31) |
|  | Guasch et al. (2016) | arousal; concreteness; valence | 23 (10, 13) |
|  | Hinojosa et al. (2016) | arousal; concreteness; valence | 6 (5, 1) |
|  | Redondo et al. (2007) | arousal; valence | 51 (20, 31) |
|  | Stadthagen-Gonzalez et al. (2017) | arousal; valence | 69 (38, 31) |
| Thai (TH) | Ngamprom et al. (2017) | valence | 19 (2, 17) |
|  | Ngamprom et al. (2017) | arousal | 5 (1, 4) |

To compare these external reliabilities with the internal split-half reliabilities, we estimated a linear mixed effects model (LMM; Bates et al., 2015; Kuznetsova et al., 2017) predicting the correlation from a fixed effect of the type of reliability (internal vs. external), plus random intercepts and random slopes by type for the rating dimension and sample. We only considered data points where at least 10 items were shared with the word norms from other studies. Here, we observed that external reliabilities were not significantly lower (*b* = -0.070, *t* = -1.846, *p* = .107) than internal split-half reliabilities (for which we observe an intercept of 0.792). We thus have no evidence to indicate that the participants performing our rating tasks diverged substantially from the participant samples from other studies. This is relevant considering that participants in our study encountered a different context with far more taboo words when providing their judgments and performed a different type of rating task (best-worst scaling instead of standard Likert scales).


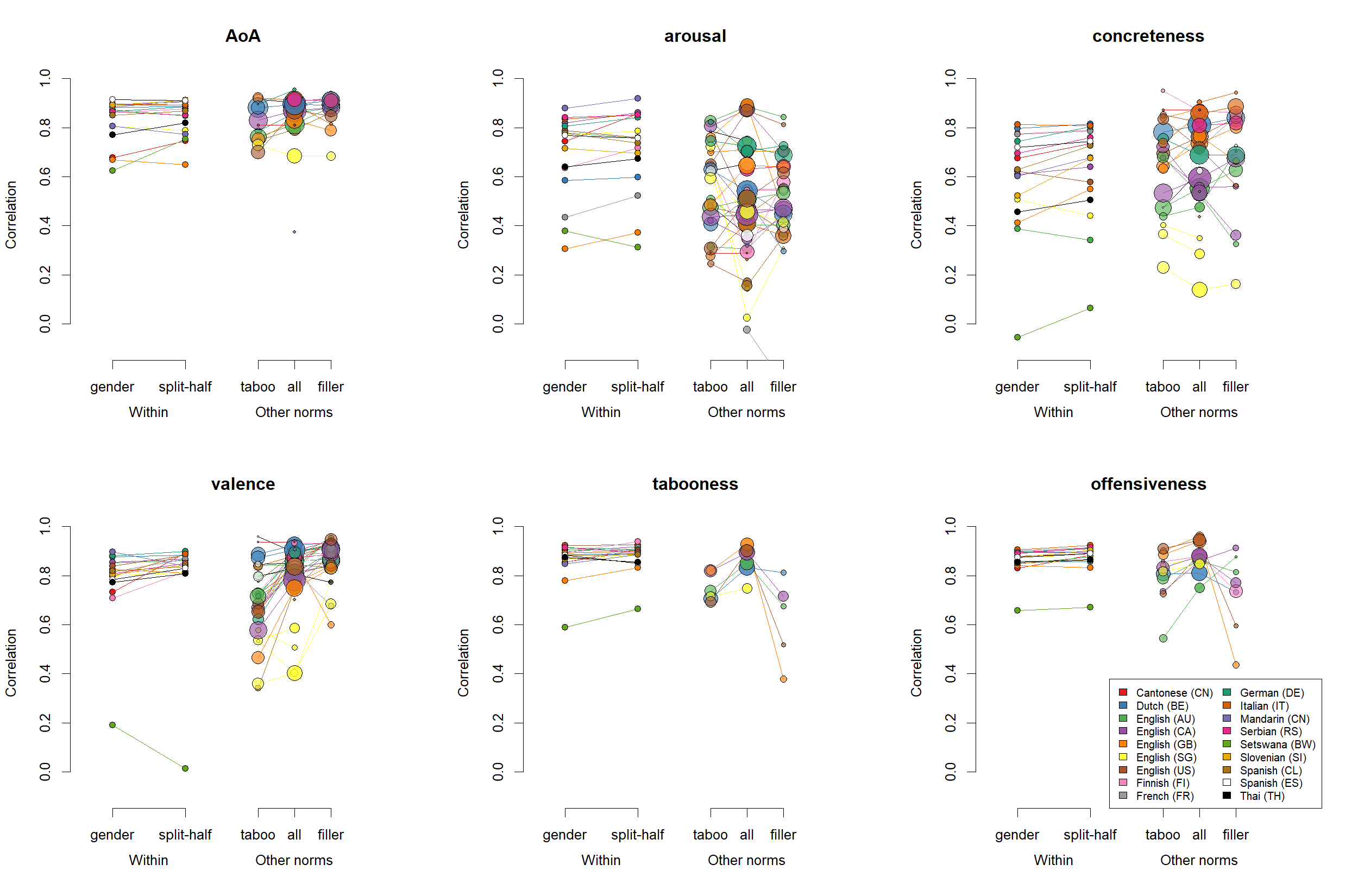


**Supplementary Figure 1**: **Internal and external reliabilities, and gender agreement.** For each sample and rating dimension, the correlation between male and female rating scores (left-most point), split-half reliability (second point from the left), and the correlation to rating scores from other word norms (right part), split by taboo words, all words, or filler words. The size of the points on the right part indicates the number of shared items (larger points = more items). Only points estimated from more than n = 10 shared items are displayed.

***The relation between valence and arousal.***

As can be seen in the top panel of Figure 5, there is indeed a U-shaped relation between valence and arousal when pooling together data from all samples. This is confirmed in a statistical analysis: Adding a quadratic fixed effect for valence to a LMM predicting arousal from a linear fixed effect for valence and a random effect for languages improves these baseline models for taboo words (*X^2^*(1) = 40.77, *p* < .001), non-taboo words (*X^2^*(1) = 130.74, *p* < .001), and both types of words combined (*X^2^*(1) = 30.25, *p* < .001). As indicated in Supplementary Figure 2, the same pattern emerges in most individual datasets too (with some noteworthy exceptions such as Mandarin, where we observe clear negative relations between valence and arousal). Note that, overall, only few taboo words tended to have very high valence ratings (in some datasets such as Cantonese, Spanish (CL), Mandarin, Serbian, English (GB), or German, such items are essentially missing entirely), so the right end of the distribution that could display a positive relation between valence and arousal is missing here.


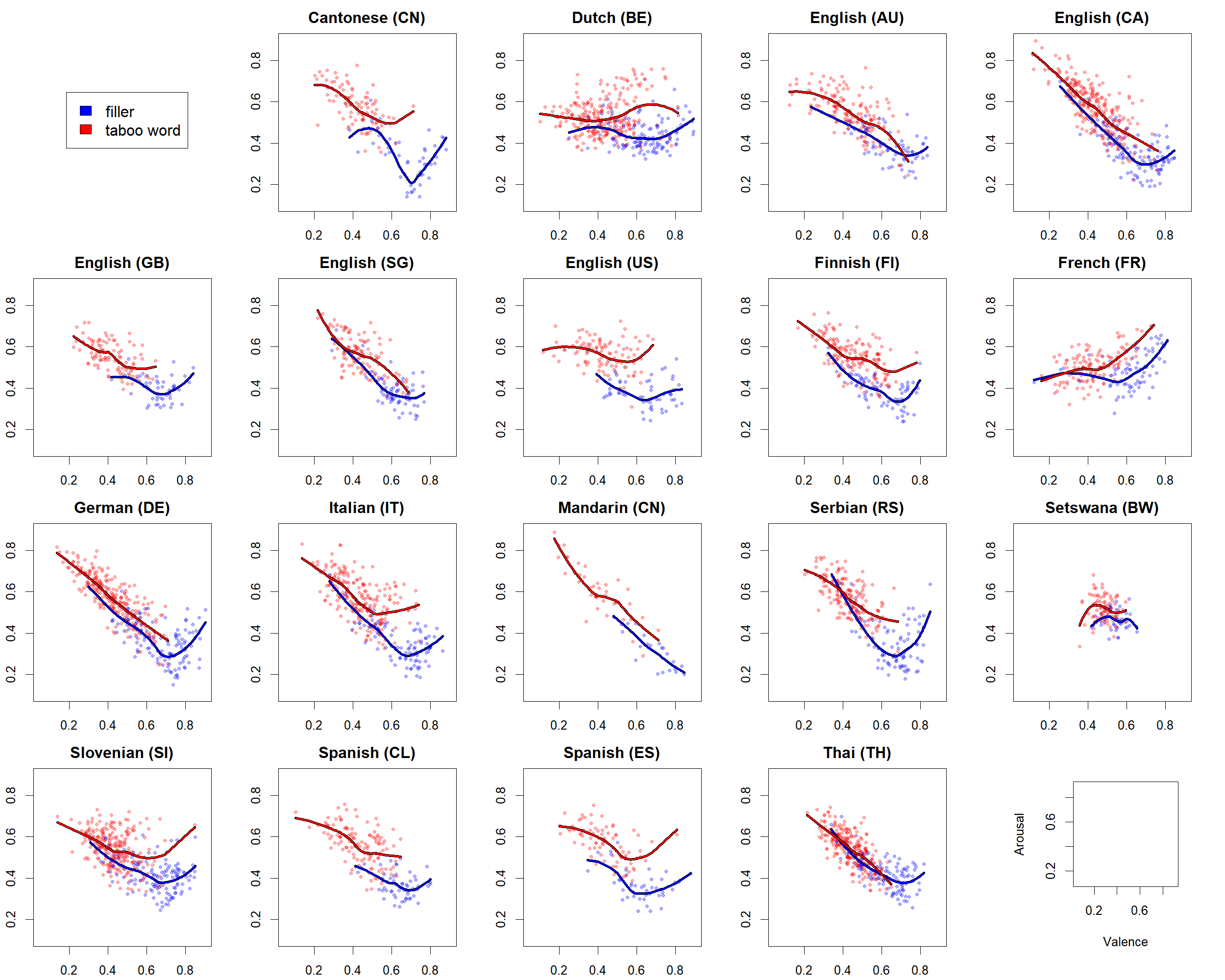


**Supplementary Figure 2: Relation between valence and arousal for taboo and filler words.** The relation between valence (x-axis) and arousal (y-axis) by sample; points indicate individual items. Note that regression lines predicting arousal from valence are fitted with local polynomial regression (loess) fitting (the loess() function in R), which display more general non-linear effects than the quadratic terms analysed here.

***Predicting tabooness and offensiveness – analysis of the taboo word subset***

Since the variables predicting tabooness/offensiveness for all items could just be the ones telling apart taboo words from non-taboo words, we repeated the mixed-effect models analysis described in the main text but restricted it to taboo words only. The results of this analysis are displayed in Supplementary Table 3. As can be seen, the general pattern of results is very similar to the analysis of all words. Therefore, the very same variables predict differences in tabooness and offensiveness ratings (and the difference between them) within the set of taboo words generated in Study 1 of this study.

**Supplementary Table 3.** Predictors of tabooness and offensiveness ratings across samples, for the dataset of taboo words (produced in Study 1) only. “Dummy” is a dummy variable encoding for tabooness ratings (coded as 0, the reference condition) or offensiveness ratings (coded as 1); therefore, the intercept and main effects except “dummy” describe tabooness ratings, while the “dummy” effect and all interactions describe how offensiveness ratings differ from tabooness ratings.

| predictor type | predictor | *b* | *t* | *p* |
| --- | --- | --- | --- | --- |
| intercept | intercept | 0.348 | 25.34 | < .001 |
| main effects | dummy | 0.414 | 21.65 | < .001 |
|  | valence | -0.388 | -29.83 | < .001 |
|  | arousal | 0.415 | 27.00 | < .001 |
|  | concreteness | 0.138 | 10.83 | < .001 |
|  | AoA | 0.219 | 21.27 | < .001 |
|  | corpus freq. | -0.006 | -5.18 | < .001 |
| interactions | dummy : valence | -0.271 | -14.74 | < .001 |
|  | dummy : arousal | -0.100 | -4.62 | < .001 |
|  | dummy : concreteness | -0.211 | -11.80 | < .001 |
|  | dummy : AoA | -0.252 | -17.44 | < .001 |
|  | dummy : corpus freq. | -0.004 | -2.46 | .014 |

**References**

Alonso, M. A., FernAndez, A., & Díez, E. (2015). Subjective age-of-acquisition norms for 7,039 Spanish words. *Behavior Research Methods*, *47*, 268-274.

Bates, D., Mächler, M., Bolker, B. & Walker, S. (2015). Fitting linear mixed-effects models using lme4. *Journal of Statiscal Software,* *67*, 1-4.

Birchenough, J. M., Davies, R., & Connelly, V. (2017). Rated age-of-acquisition norms for over 3,200 German words. *Behavior Research Methods*, *49*, 484-501.

Bonin, P., Méot, A., & Bugaiska, A. (2018). Concreteness norms for 1,659 French words: Relationships with other psycholinguistic variables and word recognition times. *Behavior Research Methods*, *50*, 2366-2387.

Bradley, M. M., & Lang, P. J. (1999). *Affective norms for English words (ANEW): Instruction manual and affective ratings* (Vol. 30, No. 1, pp. 25-36). Technical report C-1, the center for research in psychophysiology, University of Florida.

Brysbaert, M., Stevens, M., De Deyne, S., Voorspoels, W., & Storms, G. (2014). Norms of age of acquisition and concreteness for 30,000 Dutch words. *Acta Psychologica*, *150*, 80-84.

Brysbaert, M., Warriner, A. B., & Kuperman, V. (2014). Concreteness ratings for 40 thousand generally known English word lemmas. *Behavior Research Methods*, *46*, 904-911.

Cai, Z. G., Huang, S., Xu, Z., & Zhao, N. (2022). Objective ages of acquisition for 3300+ simplified Chinese characters. *Behavior Research Methods*, *54*, 311-323.

Della Rosa, P. A., Catricalà, E., Vigliocco, G., & Cappa, S. F. (2010). Beyond the abstract—concrete dichotomy: Mode of acquisition, concreteness, imageability, familiarity, age of acquisition, context availability, and abstractness norms for a set of 417 Italian words. *Behavior Research Methods*, *42*, 1042-1048.

Eilola, T. M., & Havelka, J. (2010). Affective norms for 210 British English and Finnish nouns. *Behavior Research Methods*, *42*, 134-140.

Eilola, T. M., & Havelka, J. (2011). Behavioural and physiological responses to the emotional and taboo Stroop tasks in native and non-native speakers of English. *International Journal of Bilingualism*, *15*, 353-369.

Ferrand, L., Bonin, P., Méot, A., Augustinova, M., New, B., Pallier, C., & Brysbaert, M. (2008). Age-of-acquisition and subjective frequency estimates for all generally known monosyllabic French words and their relation with other psycholinguistic variables. *Behavior Research Methods*, *40*, 1049-1054.

Filipović Đurđević, D., & Kostić, A. (2017). Number, relative frequency, entropy, redundancy, familiarity, and concreteness of word senses: Ratings for 150 Serbian polysemous nouns. In S. Halupka-Rešetar and S. Martínez-Ferreiro (Eds.) *Studies in Language and Mind 2* (pp. 13-77).

Guasch, M., Ferré, P., & Fraga, I. (2016). Spanish norms for affective and lexico-semantic variables for 1,400 words. *Behavior Research Methods*, *48*, 1358-1369.

Hinojosa, J. A., Martínez-García, N., Villalba-García, C., Fernández-Folgueiras, U., Sánchez-Carmona, A., Pozo, M. A., & Montoro, P. R. (2016). Affective norms of 875 Spanish words for five discrete emotional categories and two emotional dimensions. *Behavior Research Methods*, *48*, 272-284.

Hollis, G., Westbury, C., & Lefsrud, L. (2017). Extrapolating human judgments from skip-gram vector representations of word meaning. *Quarterly Journal of Experimental Psychology*, *70*(8), 1603-1619.

Hutton, C. & Bolton, K. *Dictionary of Cantonese slang: The language of Hong Kong movies, street gangs and city life.* (University of Hawaii Press, 2005).

Janschewitz, K. (2008). Taboo, emotionally valenced, and emotionally neutral word norms. *Behavior Research Methods*, *40*, 1065-1074.

Kanske, P., & Kotz, S. A. (2010). Leipzig affective norms for German: A reliability study. *Behavior Research Methods*, *42*, 987-991.

Kuperman, V., Stadthagen-Gonzalez, H., & Brysbaert, M. (2012). Age-of-acquisition ratings for 30,000 English words. *Behavior Research Methods*, *44*, 978-990.

Kuznetsova, A., Brockhoff, P. B., & Christensen, R. H. (2017). lmerTest package: tests in linear mixed effects models. *Journal of Statistical Software*, *82*, 1-26.

Lin, J., & Yao, Y. (2016). Encoding emotion in Chinese: a database of Chinese emotion words with information of emotion type, intensity, and valence. *Lingua Sinica*, *2*, 1-22.

Monnier, C., & Syssau, A. (2014). Affective norms for French words (FAN). *Behavior Research Methods*, *46*(4), 1128-1137.

Montefinese, M., Ambrosini, E., Fairfield, B., & Mammarella, N. (2014). The adaptation of the affective norms for English words (ANEW) for Italian. *Behavior Research Methods*, *46*, 887-903.

Montefinese, M., Vinson, D., Vigliocco, G., & Ambrosini, E. (2019). Italian age of acquisition norms for a large set of words (ItAoA). *Frontiers in Psychology*, *10*, 278.

Moors, A., De Houwer, J., Hermans, D., Wanmaker, S., Van Schie, K., Van Harmelen, A. L., ... & Brysbaert, M. (2013). Norms of valence, arousal, dominance, and age of acquisition for 4,300 Dutch words. *Behavior research methods*, *45*, 169-177.

Ng, H. (2006). *A study of Hong Kong Cantonese I.* (Subculture).

Ng, H. (2010). *A Study of Hong Kong Cantonese II*. (Subculture).

Ngamprom, C., Chadcham, S. & Wongupparaj, P. (2017). Development of the Affective Norms for Thai Words (THAI-ANW) Bank System. *Research Methodology in Cognitive Science,* *15*, 162-178.

Popović Stijačić, M. & Filipović Đurđević, D. Perceptual strength, concreteness, imageability, context availability, age of acquisition, familiarity, emotional valence, and arousal ratings for 2100 Serbian nouns and their effect on visual lexical decision latencies.

Redondo, J., Fraga, I., Padrón, I., & Comesaña, M. (2007). The Spanish adaptation of ANEW (affective norms for English words). *Behavior Research Methods*, *39*, 600-605.

Roest, S. A., Visser, T. A., & Zeelenberg, R. (2018). Dutch taboo norms. *Behavior Research Methods*, *50*, 630-641.

Schmidtke, D. S., Schröder, T., Jacobs, A. M., & Conrad, M. (2014). ANGST: Affective norms for German sentiment terms, derived from the affective norms for English words. *Behavior Research Methods*, *46*, 1108-1118.

Schröder, A., Gemballa, T., Ruppin, S., & Wartenburger, I. (2012). German norms for semantic typicality, age of acquisition, and concept familiarity. *Behavior Research Methods*, *44*, 380-394.

Söderholm, C., Häyry, E., Laine, M., & Karrasch, M. (2013). Valence and arousal ratings for 420 Finnish nouns by age and gender. *PloS One*, *8*, e72859.

Stadthagen-Gonzalez, H., Imbault, C., Pérez Sánchez, M. A., & Brysbaert, M. (2017). Norms of valence and arousal for 14,031 Spanish words. *Behavior Research Methods*, *49*, 111-123.

Su, I. F., Yum, Y. N., & Lau, D. K. Y. (2022). Hong Kong Chinese character psycholinguistic norms: Ratings of 4376 single Chinese characters on semantic radical transparency, age-of-acquisition, familiarity, imageability, and concreteness. *Behavior Research Methods*, 1-20.

Warriner, A. B., Kuperman, V., & Brysbaert, M. (2013). Norms of valence, arousal, and dominance for 13,915 English lemmas. *Behavior Research Methods*, *45*, 1191-1207.

Xu, X., & Li, J. (2020). Concreteness/abstractness ratings for two-character Chinese words in MELD-SCH. *PloS One*, *15*, e0232133.

Xu, X., Li, J., & Chen, H. (2022). Valence and arousal ratings for 11,310 simplified Chinese words. *Behavior research methods*, *54*, 26-41.

Yao, Z., Wu, J., Zhang, Y., & Wang, Z. (2017). Norms of valence, arousal, concreteness, familiarity, imageability, and context availability for 1,100 Chinese words. *Behavior Research Methods*, *49*, 1374-1385.

Yee, L. T. (2017). Valence, arousal, familiarity, concreteness, and imageability ratings for 292 two-character Chinese nouns in Cantonese speakers in Hong Kong. *PloS One*, *12*, e0174569.

1. Information about participants gender and age were not collected for Dutch as requested by the local ethical committee to increase anonymity. [↑](#footnote-ref-1)
